# Phytochemical Characterization and Antioxidant Activity Evaluation for Some Plant Extracts in Conjunction with Pharmacological Mechanism Prediction: Insights into Potential Therapeutic Applications in Dyslipidemia and Obesity

**DOI:** 10.3390/biomedicines12071431

**Published:** 2024-06-27

**Authors:** Elena Iuliana Ilie, Liliana Popescu, Emanuela-Alice Luță, Andrei Biță, Alexandru Radu Corbu, Dragoș Paul Mihai, Ana Corina Pogan, Teodora Dalila Balaci, Alexandru Mincă, Ligia Elena Duțu, Octavian Tudorel Olaru, Rica Boscencu, Cerasela Elena Gîrd

**Affiliations:** 1 Faculty of Pharmacy, University of Medicine and Pharmacy “Carol Davila”, Traian Vuia 6, 020956 Bucharest, Romania; elena.ionita@drd.umfcd.ro (E.I.I.); emanuela.luta@umfcd.ro (E.-A.L.); corina.ionita@umfcd.ro (A.C.P.); teodora.balaci@umfcd.ro (T.D.B.); ligia.dutu@umfcd.ro (L.E.D.); octavian.olaru@umfcd.ro (O.T.O.); rica.boscencu@umfcd.ro (R.B.); cerasela.gird@umfcd.ro (C.E.G.); 2 Department of Pharmacognosy & Phytotherapy, Faculty of Pharmacy, University of Medicine and Pharmacy of Craiova, Petru Rareș 2, 200349 Craiova, Romania; andrei.bita@umfcv.ro; 3 Department of Horticulture & Food Science, University of Craiova, AI Cuza 13, 200585 Craiova, Romania; alexandru.corbu@edu.ucv.ro; 4 Department of Medical Semiology, Discipline of Internal Medicine I and Nephrology, Faculty of Medicine, University of Medicine and Pharmacy “Carol Davila”, Eroii Sanitari 8, 050474 Bucharest, Romania; alexandru.minca@umfcd.ro

**Keywords:** hypolipidemic activity, anti-obesity, free radical scavenging activity, UHPLC-MS, heatmap correlation matrix, molecular docking, molecular dynamics, carbonic anhydrase 5A

## Abstract

Lipid metabolism dysregulation can lead to dyslipidemia and obesity, which are major causes of cardiovascular disease and associated mortality worldwide. The purpose of the study was to obtain and characterize six plant extracts (ACE—*Allii cepae extractum*; RSE—*Rosmarini extractum*; CHE—*Cichorii extractum*; CE—*Cynarae extractum*; AGE—*Apii graveolentis extractum*; CGE—*Crataegi extractum*) as promising adjuvant therapies for the prevention and treatment of dyslipidemia and its related metabolic diseases. Phytochemical screening revealed that RSE was the richest extract in total polyphenols (39.62 ± 13.16 g tannic acid/100 g dry extract) and phenolcarboxylic acids (22.05 ± 1.31 g chlorogenic acid/100 g dry extract). Moreover, the spectrophotometric chemical profile highlighted a significant concentration of flavones for CGE (5.32 ± 0.26 g rutoside/100 g dry extract), in contrast to the other extracts. UHPLC-MS quantification detected considerable amounts of phenolic constituents, especially chlorogenic acid in CGE (187.435 ± 1.96 mg/g extract) and rosmarinic acid in RSE (317.100 ± 2.70 mg/g extract). Rosemary and hawthorn extracts showed significantly stronger free radical scavenging activity compared to the other plant extracts (*p* < 0.05). Pearson correlation analysis and the heatmap correlation matrix indicated significant correlations between phytochemical contents and in vitro antioxidant activities. Computational studies were performed to investigate the potential anti-obesity mechanism of the studied extracts using target prediction, homology modeling, molecular docking, and molecular dynamics approaches. Our study revealed that rosmarinic acid (RA) and chlorogenic acid (CGA) can form stable complexes with the active site of carbonic anhydrase 5A by either interacting with the zinc-bound catalytic water molecule or by directly binding Zn^2+^. Further studies are warranted to experimentally validate the predicted CA5A inhibitory activities of RA and CGA and to investigate the hypolipidemic and antioxidant activities of the proposed plant extracts in animal models of dyslipidemia and obesity.

## 1. Introduction

In line with the rapid development at the global level, more and more diverse health problems appear among the population. People do not have time anymore, and very diverse eating habits follow a downward slope, often characterized by hyperlipidemias, accompanied or not by obesity. However, although obesity can be seen with the naked eye, hyperlipidemias can be considered the silent enemy, being detectable only through biochemical analyses. Although a healthy lifestyle has begun to be increasingly sought after among people, it does not necessarily ensure health since the main factor that negatively influences all processes in the body is stress. Hyperlipidemia and obesity are complex pathological conditions that do not necessarily appear as consequences of the disease but rather predispose to the disease [1,2], especially cardiovascular diseases (stroke, myocardial infarction, atherosclerosis), arterial hypertension, cancers of various types (colon, kidney, breast), type 2 diabetes, osteoarthritis, apnea, liver and biliary diseases, and depression, with the possibility of generating other mental manifestations [3,4].

In order to keep up with the current trends in acquiring and maintaining a healthy lifestyle, adjuvants from the plant world are increasingly being used. Thus, there are extracts obtained from plant products that maintain lipid profiles at a normal level and help against oxidative stress by fighting free radicals.

The present research aims to evaluate the content of total polyphenols and the antioxidant effect induced by the extracts obtained from plant products from medicinal species that grow in Romania (sold in specialized stores in the form of medicinal teas) or that are associated with the diet. Among the potential plant sources with hypolipidemic and antiradical effects, the following were selected: *Rosmarini folium*, *Cynarae folium*, *Crataegi folium cum flos*, *Cichorii herba* (medicinal products), *Allii cepae herba*, and *Apii graveolentis herba* (food products).

Thus, extracts of rosemary (*Rosmarini extractum*), obtained by different procedures and administered to obese rats or fed with a hyperlipidic diet, decrease total cholesterol, triglycerides, and lipid content in the liver and in white and brown adipose tissues [5,6], through the action of rosmarinic and carnosic acids [7], decrease the atherogenic index [8,9,10], and reduce free fatty acids, hypertrophy, and necrosis of hepatocytes [11]. On the other hand, due to the antioxidant effect, there is the regulation of epigenetic and antiangiogenic activity [12], the blocking of AFB1 synthesis by scavenging on free radicals [13,14], the inhibition of lipid peroxidation, and the stabilization of membranes [15,16].

Regarding the chemically active compounds in artichoke, luteolin inhibits HMGCoA-reductase [17] and, together with chlorogenic acid, produces a decrease in total cholesterol, LDL-cholesterol, and triglycerides [18,19], while caffeic acid and cynarin reduce the concentration of reactive oxygen species and stimulate the activity of antioxidant enzymes [20,21,22,23].

Extracts obtained from different hawthorn species decrease triglycerides and cholesterol [24], maintain the antioxidant status of mitochondria, prevent lipid peroxidation, and decrease Krebs cycle enzymes [25]. Together with the help of oligomeric proanthocyanidins, triterpenes, flavonoids, polysaccharides, and catecholamines, they maintain the redox balance in the body [26,27].

Onion extracts administered to rats fed a hyperlipidic diet reduce total lipids, decrease plasma and liver cholesterol [28], decrease triglycerides, malonylaldehyde, and LDL cholesterol, and increase SOD activity and HDL cholesterol levels [29,30,31,32,33,34,35,36,37,38].

By luteolin, quercetin, kaempferol, apigenol, and other constituents of celery extract (Apium graveolens), decreases in total serum cholesterol, LDL cholesterol, and triglycerides are induced [39,40,41,42,43,44,45,46,47,48], and scavenger capacity on free radicals is also decreased [49,50,51,52].

Previous research on chicory (*Cichorii herba*) has shown stopping the accumulation of cholesterol in the aorta, lowering triglycerides, LDL cholesterol, and total cholesterol, regulating HMGCoA (by increasing the turnover rate of cholesterol in the blood and liver), while also having an antioxidant effect [53,54,55,56,57,58].

The use of Ultra-High Performance Liquid Chromatography (UHPLC) coupled with Mass Spectrometry (MS) allowed for a highly efficient and sensitive analysis. UHPLC offers several advantages over traditional HPLC, including faster run times, higher resolution, and greater sensitivity. When coupled with MS, it becomes a powerful tool for the identification and quantification of compounds at very low concentrations. This combination is particularly useful for polyphenols, which often require sensitive detection methods due to their diverse structures and physicochemical properties.

In this work, we aimed at obtaining the following six plant extracts with potential use as adjuvant therapy for dyslipidemia and obesity: ACE—*Allii cepae extractum*; RSE—*Rosmarini extractum*; CHE—*Cichorii extractum*; CE—*Cynarae extractum*; AGE—*Apii graveolentis extractum*; and CGE—*Crataegi extractum*. We thereafter performed phytochemical characterization by assessing the contents of polyphenolic compounds. The antioxidant activity of the characterized extracts was assessed by three in vitro methods. Lastly, in silico studies based on molecular docking and molecular dynamics simulations were performed for selected phytochemicals identified in the extracts to investigate the potential molecular mechanisms that would support the hypolipidemic and anti-obesity activities. 

## 2. Materials and Methods

### 2.1. Reagents, Chemicals, and Materials

All reagents and chemical substances of analytical grade were purchased from various approved suppliers. In brief, 2,2-diphenyl-1-picrylhydrazyl (DPPH), ABTS ammonium salt 7.4 mM, Folin–Ciocâlteu reagent, 10% trichloroacetic acid, phosphate buffer (pH = 6.6), 1% potassium hexacyanoferrate (K_3_(FeCN)_6_), 0.1% ferric chloride (FeCl_3_), potassium persulfate 2.6 mM, aluminum chloride 25 g/L, sodium acetate 100 g/L, hydrochloric acid 0.5 M, sodium nitrite, Arnow reagent, sodium hydroxide 85 g/L, and sodium carbonate solution 200 g/L were purchased from Sigma-Aldrich, Darmstadt, Germany. The ethanol used as a solvent in this study was also purchased from Sigma-Aldrich, Darmstadt, Germany.

Reference substances (100% purity) such as ascorbic acid, tannic acid, rutin, and chlorogenic acid were purchased from Sigma, Germany. 

Spectrophotometric assessment was performed using a Jasco V-530 spectrophotometer (Tokyo, Japan), and for the freeze-drying process, the equipment used was a rotary evaporator (Buchi, Vacuum Pump V-700, Thermo Fisher Scientific, Waltham, MA, USA) and a vacuum freeze-drying machine (Christ Alpha 1-2/B Braun, BiotechInt, Braun Biotech International GmbH, Melsungen, Germany).

For the sensitive analysis of the phenolics in the studied plant extracts, we used Ultra-High Performance Liquid Chromatography (UHPLC) coupled with Mass Spectrometry (MS) for the highly efficient identification and quantification of phytocompounds. The reference compounds used for the UHPLC-MS method were also obtained from Merck (Merck Romania, Bucharest, Romania) and Sigma-Aldrich, Darmstadt, Germany. Methanol and ultrapure water (UHPLC grade) were provided by Merck (Merck Romania, Bucharest, Romania). The exact origin of the standards and other experimental conditions used in each stage of the determinations were presented in the assigned section.

### 2.2. Extraction Procedure and Plant Extract Material 

To obtain the extracts, two plant products were purchased from the local pharmaceutical market in the form of single component teas (*Cynarae folium* and *Cichorii herba*, both in dried form); three plant products were purchased from Romanian producers (*Rosmarini folium* and *Crataegi folium cum flos* in dried form and *Allii cepae herba* in fresh form, for alimentary purposes); and one plant product was purchased from the supermarket in fresh form (*Apii graveolentis herba*, also for alimentary purposes). These plant products were subjected to refluxing with ethanol, concentration by rotary evaporation, and finally freeze-drying (lyophilization). Two successive refluxings of 30 min each were carried out at a ratio of plant product to solvent of 1:20 and 1:10, respectively. Two concentrations of ethanol were used, as follows: 50% ethanol for *Cichorii herba*, *Cynarae folium*, and *Crataegi folium cum flos*, respectively 70% ethanol for *Allii cepae herba*, *Rosmarini folium*, and *Apii graveolentis herba*. After obtaining the extracts, conditioning was performed in an appropriate desiccator. 

### 2.3. Phytochemical Screening by Spectrophotometric Methods

#### 2.3.1. Assessing the Concentration of Flavones

We applied the previously described methods [59] using the property of flavonoid compounds to react in the presence of trivalent metals (AlCl_3_) with the formation of intense yellow chelates. The samples were read against the corresponding controls at 427 nm after 45 min. In order to determine the flavone content, tests were conducted on variable volumes of sample, from 1 mL to 1.8 mL, for all the extracts, with a rate of progression of 0.2 mL. These analyses were expressed as g rutin equivalents per gram of sample (g/g), and rutin (rutoside) was used as a standard for the linear calibration curve.

#### 2.3.2. Evaluation of Total Polyphenol Content

For plant extract samples, total polyphenol content was determined using the well-established Folin–Ciocâlteu method [60]. The absorbance of prepared samples was measured at 763 nm against a blank after 40 min in dark conditions. For the evaluation of total polyphenol content, varying volumes of sample were taken into testing, between 0.4 mL and 0.8 mL, with a rate of progression of 0.1 mL. Total polyphenol content was expressed as g tannic acid equivalents per gram of sample (g/g).

#### 2.3.3. Phenolic Acid Content Assessment

Phenolcarboxylic acids, or phenolic acids, were determined spectrophotometrically using the Arnow reagent as a color reagent (red samples compared to yellow controls) [61]. The absorbance was immediately measured at 525 nm using an UV-VIS JASCO V-530 spectrophotometer. For the phenolic acid content evaluation, tests were conducted on variable volumes of sample, from 0.8 mL to 1.6 mL, for all the extracts, with a rate of progression of 0.2 mL. Results were expressed in g chlorogenic acid equivalents per gram of sample (g/g).

### 2.4. UHPLC-MS Method

Before being injected, the working samples were made from a solubilization of 10 mg of plant extract dissolved in 5 mL of 70% methanol. An ACQUITY Arc System with an ACQUITY QDa detector was used to perform the separation of samples, which were filtered through 0.2 μm syringe filters (Acrodisc MS Syringe Filters WWPTFE Membrane). A CORTECS C18 column with the following specifications—length of 50 mm, an inside diameter of 4.6 mm, and particle sizes of 2.7 μm—was used to achieve LC separation. Formic acid 0.1% (solvent A) and acetonitrile (solvent B) constituted the gradient mobile phase, which had a flow rate of 0.8 mL/min. The injection volume of the sample was 5 µL. The samples were maintained at 10 °C while the separation process was run at a column temperature of 28 °C. The following parameters were used for Mass Spectrometry analysis: negative ion mode, a cone voltage of 20 V, a capillary voltage of 0.8 kV at a source temperature of 120 °C, and a disintegration temperature of 400 °C. We processed and acquired data using the Empower 3 software. Mass spectra were detected using the following m/z: 153, 163, 167, 169, 179, 193, 197, 263, 301, 353, and 359, with a sample rate of 10 points/sec. The reference preparation, the characteristics of each compound’s calibration curve, and the mobile phase gradient are explained in detail in Supplementary Materials Tables S1 and S2.

### 2.5. Evaluation of Antioxidant Potential

#### 2.5.1. DPPH-Free Radical Scavenging Assay

The free radical scavenging ability of the extracts against DPPH (2,2-diphenyl-1-picrylhydrazyl) was carried out according to the previously described technique [62,63]. Volumes ranging from 100 μL to 1000 μL were taken for all determinations, with increments of 100 μL between samples. Ascorbic acid was used as the antioxidant reference. The scavenging ability of the extracts was calculated using the IC50 value (mg/mL).

#### 2.5.2. Antioxidant Activity by the ABTS^+^ Method

This antioxidant in vitro method is based on the scavenging of ABTS^∙+^ from antioxidants in the vegetal sample, which produce a spectrophotometric loss in absorbance at 734 nm. The complete experimental protocol was presented in our previous research work [60,64]. Briefly, volumes ranging from 100 μL to 1000 μL were taken for all determinations, with increments of 100 μL between samples. The IC50 values for each plant extract were calculated from the plotted graph of radical scavenging activity against the concentration of extracts (mg/mL).

#### 2.5.3. Reducing Ferric Capacity

The ferric reducing assay emphasizes the ability of the analyzed extracts to reduce ferric iron to ferrous iron with the appearance of a specific blue color. According to our previous study, we used the same experimental steps as were described [65]. The absorbance for each sample was measured at 700 nm. Concisely, volumes ranging from 100 μL to 1000 μL were taken for all samples, with a rate of progression of 100 μL. The final antioxidant capacity was determined using the EC50 value (mg/mL). 

### 2.6. Statistical Analysis

Statistical analysis of the phytochemical composition and antioxidant results was performed using a one-way analysis of variance (ANOVA), followed by post hoc analysis with the Games–Howell test for unequal variances (*α* < 0.05), using the SPSS Statistics version 29.0.2.0 (20) software package (IBM Corporation, Chicago, IL, USA). The F-values and degree of freedom (*df*) of the statistical analyses were also calculated. Multiple comparisons of the data were performed to compute significant differences at *p* < 0.05. Comparisons between groups and within groups were analyzed, and descriptive statistics and a 95% confidence interval were run for different data sets. Normality tests (Kolmogorov–Smirnov test and Shapiro–Wilk test), Levene’s test for the homogeneity of variances, and robust tests of equality of means (Welch *t*-test and Brown–Forsythe’s test) were implemented to assess and verify data analysis assumptions. 

When some mandatory assumptions were violated or broken, data transformation was used (reciprocal, square root, reciprocal-square root) in order to be able to draw accurate conclusions about reality after subjecting the data to parametric statistical tests.

Pearson’s correlation coefficient analysis was calculated using the SPSS program in order to evaluate the statistical relationship between phytochemical compounds and antioxidant activities. Normal Q-Q plots, histograms, heatmap correlation matrix, and pyramid models were drawn up, which were very useful in interpreting the results. The results were expressed as means and standard deviations for at least three replications for each prepared sample. The level of statistical significance was established at a *p*-value < 0.05.

### 2.7. Computational Studies

A computational workflow was implemented to identify potential molecular mechanisms for the phytochemicals contained in the investigated plant extracts to further warrant their potential use in managing obesity and dyslipidemia.

#### 2.7.1. Target Prediction

The phytoconstituents identified in the highest concentrations in the investigated plant extracts were selected for further computational studies. The biological activities of the selected phytochemicals were predicted using several freely available web servers, i.e., PASS (Prediction of Activity Spectra for Substances), SwissTargetPrediction, and SEA (Similarity Ensemble Approach). PASS server is a machine learning algorithm that uses Level 2 Multilevel Neighborhoods of Atom descriptors as independent variables. The predictive algorithm is based on a Bayesian approach and estimates for input ligands the probability of being active (Pa) or inactive (Pi) on a specific target, or the probability of having a specific pharmacological activity [66]. SwissTargetPrediction is another tool that predicts the probability of modulating specific target proteins based on a combination of 2D and 3D similarity measures between the input ligands and known active molecules [67]. SEA is a computational approach that relates proteins based on the chemical similarity of known actives and calculates Tanimoto similarity scores (Tc) for each query ligand using 2D topological Daylight fingerprints [68]. Isomeric SMILES codes of the selected ligands were retrieved from the PubChem database (Supplementary Materials Table S3) [69] and were used as input variables for each method. Results were manually filtered to select key therapeutic targets with potential roles in treating obesity and dyslipidemia [70,71].

#### 2.7.2. Homology Modeling

The target prediction approaches identified carbonic anhydrase 5A (CA5A) as a potential therapeutic target for the selected phytochemicals. Considering that there are no readily available crystal structures of human CA5A, we opted for homology modeling methods to build tridimensional models of the human protein. The amino acid sequence of Ca5A was retrieved from the UniProt database (code P35218) in FASTA format. YASARA Structure [72] software (version v22.5.22) is a fully automated tool that was used to build protein models. Templates were manually selected based on existing crystal structures of mouse CA5A. To this extent, we chose two PDB structures, i.e., one that contained a catalytic water molecule bound to Zn^2+^ and a covalent modification with 4-chloromethylimidazole (PDB ID: 1KEQ, 1.88 Å [73]), and another structure with an inhibitor (acetazolamide) directly bound to the catalytic Zn^2+^ (PDB ID: 1DMY, 2.45 Å [74]). The quality of the generated models was assessed using the MolProbity web server [75,76]. The assessed quality parameters included the clash score (number of clashes per 1000 atoms), poor rotamers (percentage of side chains in statistically uncommon/unfavorable rotamer conformations), favored rotamers (percentage of side chains that are in favorable conformations), Ramachandran outliers (residues that do not fall within regions typically observed in high-resolution structures with respect to the backbone conformation), Ramachandran favored (residues with backbone conformations in the most favored regions of the Ramachandran plot), Z-score (a comparison of the model overall quality with scores associated with high-quality structures; more negative is better), MolProbity score (a combined quality measure; closer to 1 is better), Cβ deviations (deviations of β carbons from ideal positions), bad bonds (discrepancies in bond lengths when compared to ideal values), bad angles (discrepancies in bond angles compared to ideal values).

#### 2.7.3. Molecular Docking

Molecular docking simulations were carried out to predict the potential interactions between the selected ligands and the two modeled conformations of human CA5A. The docking studies were performed with the AutoDock Vina v1.1.2 [77] algorithm within YASARA Structure in a directed manner. The grid box (20 × 20 × 20 Å) was set around the active site, which includes the catalytic Zn^2+^ (X = 2.62771 Å, Y = 0.35508 Å, and Z = −3.09498 Å for the 1KEQ homologue; X = 1.92791 Å, Y = −0.71868 Å, and Z = 3.70724 Å for the 1DMY homologue) complexed by three histidine residues (His130, His132, and His155) and a water molecule (in the case of the model based on the 1KEQ structure). Other residues that form the binding pocket include the proton shuffle residue Tyr167, residue Thr235, which is typically involved in binding the phenolic CA inhibitors [78], Glu142, which forms an intramolecular hydrogen bond with Thr235, polar residues Thr236, Gln103, and Gln128, and hydrophobic residues Val157, Val171, Val177, Val179, Leu234, Val243, and Trp245, which delimit the binding cavity. Two known CA5A inhibitors were also docked into the binding site for comparison as positive controls—caffeic acid (CFA) [79] for the model, which includes the zinc-bound water molecule, and acetazolamide for the model, which was built based on the complex between acetazolamide and mouse CA5A.

Protein and ligand structures were protonated according to the physiological pH (7.4). The 3D ligand structures were energetically minimized using MMFF94s+ forcefield. Docking was performed with rigid side chains, with 12 runs for each ligand; the results were retrieved as the binding energy (ΔG, kcal/mol). The predicted binding poses and molecular interactions were analyzed using BIOVIA Discovery Studio Visualizer (BIOVIA, Discovery Studio Visualizer, Version 17.2.0, Dassault Systèmes, 2016, San Diego, CA, USA).

#### 2.7.4. Molecular Dynamics

Molecular dynamics (MD) simulations were performed to further evaluate the docking results and to analyze the stability of the predicted protein–ligand complexes. The ligand-free (apo) structure of the target protein was also simulated for reference (negative control), and the complexes between CA5A and CA or AZM were simulated as positive controls. Simulations were carried out using YASARA Structure for a total duration of 100 ns. First of all, the hydrogen bonding network was optimized to increase stability [80]. The system was neutralized by adding NaCl ions at 0.9% concentration. Clashes were removed using steepest descent and simulated annealing minimizations. The following force fields were used for each component of the system: AMBER14 for the protein structure [81], GAFF2 [82] and AM1BCC [83] for ligand atoms, and TIP3P for water molecules. A cut-off of 8 Å was used for van der Waals forces [84]. Particle mesh Ewald method with no cut-off was used for long-range electrostatic calculations [85]. Simulations were carried out at 298 K and 1 atm (NPT ensemble). The integration of motion equations was performed with a multiple timestep of 5 fs for non-bonded and 2.5 fs for bonded interactions [86]. The free energies of binding (kcal/mol) were calculated for the simulated phytochemicals using the Poisson–Boltzmann (MM/PBSA) approach, excluding the entropic term [87].

## 3. Results

### 3.1. Phytochemical Analysis

Phytochemical analysis revealed important amounts of secondary metabolites, especially total polyphenols, phenolic acids, and flavones, for each analyzed extract.

Due to their molecular structure, which is characterized by numerous hydroxyl groups, phenolic compounds have a high potential to neutralize free radicals [88,89]. 

The phytochemical composition was investigated using the spectrophotometric method in order to quantify each class of compounds. Our phytochemical results are summarized in Table 1 and in Figure S1a–c (Supplementary Materials) for the analyzed extracts, i.e., ACE—*Allii cepae extractum*; RSE—*Rosmarini extractum*; CHE—*Cichorii extractum*; CE—*Cynarae extractum*; AGE—*Apii graveolentis extractum*; CGE—*Crataegi extractum*. The extraction yield for the obtained extracts was as follows: 5.92% for ACE, 32.22% for RSE, 30.56% for CHE, 42.69% for CE, 5.70% for AGE, and 24.46% for CGE.

Our findings emphasized that the highest TP concentrations for RSE (39.62 ± 13.16 g tannic acid/100 g dry extract), CHE (30.51 ± 1.96 g tannic acid/100 g dry extract), and CGE (25.93 ± 1.10 g tannic acid/100 g dry extract) were statistically significant (*p* < 0.05) compared to the ones obtained for the other extracts. A significant difference among the plant extracts was also observed for the PCA determination (*p* < 0.05), revealing higher values for RSE (22.05 ± 1.31 g chlorogenic acid/100 g dry extract) and CGE (14.05 ± 1.65 g chlorogenic acid/100 g dry extract) as well. The chemical profile of FL showed that hawthorn extract was the richest, with a significant concentration of 5.32 ± 0.26 g expressed in rutoside (*p* < 0.001).

The plant extract with the lowest concentration of phytocompounds was AGE (FL: 0.35 ± 0.07 g rutoside/100 g dry extract; TP: 0.57 ± 0.06 g tannic acid/100 g dry extract; PCA: 0.38 ± 0.01 g chlorogenic acid/100 g dry extract). Modest but statistically significant concentrations of FL (2.03 ± 0.12 g rutoside/100 g dry extract) and TP (3.77 ± 0.84 g tannic acid/100 g dry extract) were recorded for ACE, while the level of PCA in the onion extract could not be detected spectrophotometrically.

### 3.2. UHPLC-MS Polyphenol Assay

The UHPLC-MS method employed a gradient elution technique, starting with a higher concentration of a polar solvent and gradually increasing the proportion of a less polar solvent to separate compounds based on their polarity. This method is particularly effective for analyzing complex mixtures of polyphenols, which vary in their solubility and chemical properties. The Mass Spectrometry (MS) component provided the capability to precisely identify the compounds based on their mass-to-charge ratios, offering detailed insights into the chemical composition of each sample.

A total of six samples were analyzed, each for its polyphenolic content. This selection provided a comparative view of the phytochemical diversity present in different plant materials and their potential health benefits.

The analysis led to the identification of several key polyphenols, including chlorogenic acid, rosmarinic acid, protocatechuic acid, caffeic acid, and syringic acid. These compounds were quantified in six different extracts, each representing a specific type of plant material, such as *Cynarae extractum* (artichoke leaf), *Apii graveolentis extractum* (celery herb), *Allii cepae extractum* (scallion herb), *Cichorii extractum* (chicory herb), *Crataegi extractum* (hawthorn leaf with flowers), and *Rosmarini extractum* (rosemary leaf).

For better visibility, the results can be seen in Supplementary Materials Figure S2a–f, where the spectra of the chemical compounds identified for each type of plant extract analyzed are presented, and in Table 2 are shown their quantifications in mg/g.

The chromatogram of *Allium cepa* (Supplementary Materials Figure S2c) has been rescaled to enhance the visibility of various compound peaks, particularly those at lower concentrations. This rescaling is essential for a more detailed analysis, as it allows for the differentiation and accurate identification of compounds present in smaller amounts. Additionally, the chromatogram of ACE shows a significant baseline drift for the channel monitoring m/z 179, which is indicative of caffeic acid. This drift suggests the presence of a peak with little to no retention time on the column being used. Such peaks are typically eluted almost immediately after sample injection and can be attributed to compounds that do not interact with the stationary phase of the column. This can be a common occurrence with highly polar compounds in certain chromatographic conditions, or it could signify the presence of an unretained substance within the matrix.

The main compounds found across all samples with respect to their quantity, based on the data obtained through the UHPLC-MS analysis, are: chlorogenic acid (S1, S5), rosmarinic acid (S6), protocatechuic acid (S1, S4, S5), caffeic acid (S1, S4, S6), and syringic acid (S1–S6).

The S3 sample (*Allii cepae* extract), obtained from the scallion herb, exhibits a lower polyphenol concentration compared to the other samples analyzed. Polyphenol content in plant extracts can vary widely due to numerous factors that impact phytochemical composition, including the specific part of the plant analyzed, growth conditions, post-harvest handling, processing, and extraction methods.

Chlorogenic acid was isolated in a huge concentration in the S5 sample (CGE: 187.435 ± 1.96 mg/g extract), whereas in onion extract (S3) and rosemary extract (S6) it was detected under the quantification limit. A considerable amount of chlorogenic acid was observed in the S1 sample (CE: 78.529 ± 1.15 mg/g extract).

Rosmarinic acid was quantified in small ranges (0.039 ± 0.01–0.095 ± 0.01) in each analyzed extract, but the largest amount was found in RSE (S6: 317.100 ± 2.70 mg/g). 

Vanillic acid, detected in concentrations ranging from 0.748 ± 0.069 to 1.782 ± 0.067 mg/g across the samples (S1 to S6), is a derivative of benzoic acid known for its antioxidant properties. It contributes to the neutralization of free radicals and the prevention of lipid peroxidation, thus protecting cellular components from oxidative damage.

Ferulic acid, with concentrations found from 0.055 ± 0.010 to 0.265 ± 0.024 mg/g in the samples, is a hydroxycinnamic acid derivative recognized for its strong antioxidant capacity. It is particularly effective in stabilizing lipid membranes and preventing the oxidation of low-density lipoproteins (LDL), a key factor in the development of atherosclerosis.

### 3.3. Free Radical Scavenging Activity of Vegetal Extracts

The specific antioxidant capacity provides valuable information about the effectiveness of phenolic classes to neutralize free radicals. The concentration of phenolic compounds and several characteristics of their chemical structure (their donor-proton capacity, the number and position of hydroxyl groups, the presence of glycosylations, or the electron delocalization of the aromatic nucleus) are strongly related to the antioxidant activity of the analyzed extracts [88,90].

The mixture of these antioxidant principles present in extracts can generate distinct supplementary effects, such as additively, antagonistically, and synergistically, that influence the total capacity of the extracts to annihilate oxidative stress [88,90].

In our research, scavenging activity was evaluated by three in vitro well-known methods, i.e., ABTS assay, DPPH assay, and FRAP assay, based on the remarkable reactivity of free radicals DPPH^•^ (2,2-diphenyl-picryl-hydrazyl) and ABTS^•^ (2,2-azinobis-3-ethylbenzotiazoline-6-sulfonic acid), but also based upon ferric ion reducing activity.

#### 3.3.1. Analysis of Differences between Antioxidant Activities

A one-way ANOVA analysis was performed to compare the antioxidant effects induced by the tested plant extracts, evaluating the statistical significance of the differences between the inhibitions of free radical activity developed by these extracts.

The statistical evaluation was carried out for all three methods of determining the antioxidant activity (DPPH, ABTS, and FRAP), and in order to give a more complete picture of the data sets, a descriptive statistics table was run for each set, which shows the mean and standard deviation of the inhibition values induced by every vegetal extract group (Supplementary Materials Tables S4–S6). Normal Q-Q plots for each vegetal extract group from every antioxidant method are presented in Supplementary Materials Figures S3–S5.

Using the DPPH method, the one-way ANOVA (Supplementary Materials Table S7) revealed that there was a statistically significant difference in antioxidant scavenging activity between at least two groups (F(5, 54) = [12.029], *p* < 0.001). After the application of the robust tests (Supplementary Materials Table S8), but also according to the ANOVA analysis, the difference is highly significant at the significance threshold of 99% (*p* < 0.001). 

So, after multiple comparisons of means between the groups for the DPPH method with the post hoc tests (Supplementary Materials Table S9), there is a statistically significant difference between *Allium_cepa* vs. *Rosmarinus* (*p* = 0.004, 95% C.I. = [0.118, 0.576]), *Allium_cepa* vs. *Cichorium (p =* 0.002, 95% C.I. = [0.087, 0.363]), *Allium_cepa* vs. *Cynara* (*p =* 0.011, 95% C.I. = [0.043, 0.326]), *Crataegus* vs. *Allium_cepa* (*p* = 0.006, 95% C.I. = [0.110, 0.603]*)*, *Rosmarinus* vs. *Apium* (*p* = 0.005, 95% C.I. = [0.108, 0.566]), *Cichorium* vs. *Apium* (*p =* 0.003, 95% C.I. = [0.076, 0.353]), *Cynara* vs. *Apium* (*p* = 0.016, 95% C.I. = [0.032, 0.316]), and *Apium* vs. *Crataegus* (*p* = 0.007, 95% C.I. = [0.099, 0.593]). The confidence interval (95% C.I.) is reported as the range of absolute values.

The antioxidant capacities assessed using the ABTS method were compared with the ANOVA test and robust tests (Welch test and Brown–Forsythe test) in order to see if the scavenging effect differs significantly between plant extracts (Supplementary Materials Tables S10 and S11). A statistically significant difference was found, expressed by F(5, 36) = [11.316], *p* < 0.001. 

Because it is not known exactly between which plant groups there are significant differences, the Games–Howell test was run to evaluate multiple comparisons. 

The differences between the groups are quantified in Supplementary Materials Table S12 as follows: *Allium_cepa* vs. *Rosmarinus* (*p* = 0.013, 95% C.I. = [0.062, 0.507]), *Allium_cepa* vs. *Cichorium* (*p* = 0.048, 95% C.I. = [0.002, 0.522]), *Crataegus* vs. *Allium_cepa* (*p* = 0.016, 95% C.I. = [0.077, 0.682]), *Rosmarinus* vs. *Apium* (*p* = 0.002, 95% C.I. = [0.159, 0.605]), *Cichorium* vs. *Apium* (*p* = 0.009, 95% C.I. = [0.099, 0.619]), and *Apium* vs. *Crataegus* (*p* = 0.004, 95% C.I. = [0.175, 0.779]). The confidence interval (95% C.I.) is reported as the range of absolute values.

Following the FRAP antioxidant method, we obtained a statistically significant overall *p*-value of the ANOVA assay (F(5, 54) = [15.897], *p* < 0.001), which confirms the existence of statistical differences between extracts (Supplementary Materials Tables S13 and S14). 

Therefore, post hoc multiple comparisons for unequal variances were performed to detect the pairs of plant extracts whose antioxidant effects differed significantly. The Games–Howell test (unequal variances) found statistically significant differences between the analyzed extracts (Supplementary Materials Table S15) in terms of optical density obtained as follows: *Allium_cepa* vs. *Rosmarinus* (*p* = 0.011, 95% C.I. = [0.059, 0.459]), *Cichorium* vs. *Allium_cepa* (*p* = 0.000, 95% C.I. = [0.150, 0.491]), *Allium_cepa* vs. *Cynara* (*p* = 0.000, 95% C.I. = [0.380, 0.617]), *Apium* vs. *Allium_cepa* (*p* = 0.000, 95% C.I. = [0.151, 0.297]), *Crataegus* vs. *Allium_cepa* (*p* = 0.002, 95% C.I. = [0.145, 0.547]), *Rosmarinus* vs. *Cynara* (*p* = 0.024, 95% C.I. = [0.026, 0.452]), *Cynara* vs. *Apium* (*p* = 0.000, 95% C.I. = [0.148, 0.401]). The confidence interval (95% C.I.) is reported as the range of absolute values.

#### 3.3.2. Pearson Assay and Heatmap Correlation Matrix

A Pearson correlation assay was conducted because this statistical tool can highlight if there is a possible relationship between the concentration of chemical compounds and the exerted antioxidant effect. Thus, the correlation coefficient (Pearson’s coefficient r) evaluates the nature of the connection between the analyzed data sets and can tell us whether two variables move in the same or opposite direction. It also indicates how strong the relationship is, and its value ranges from −1 to 1.

Bioactive compounds from plant extracts, such as total phenolic content, flavonoids, or phenolcarboxylic acids, can exhibit important biological effects and promising antioxidant activity owing to their capability to scavenge reactive oxygen species effectively. In this manner, the need to quantify the contribution of these active principles to the manifestation of the antioxidant effect in vitro or in vivo currently remains an essential part of research.

Our statistical studies demonstrated that the in vitro antioxidant activities and the phytochemical content of the analyzed extracts were correlated. The database for the Pearson assessment is illustrated in the Supplementary Materials Table S16.

Correlation analysis emphasizes the strength of data association for different significance levels (0.01 level and 0.05 level), the outcomes being presented in Supplementary Materials Table S17.

The Pearson coefficient between the antioxidant effect expressed as the IC50 value (ABTS method) and total polyphenols was −0.825 (*p* = 0.043, *p* < 0.05), revealing a strong inverse relationship that indicated that the antioxidant effect was likely contributed mainly by polyphenolic compounds. 

A similar result of a correlation coefficient of −0.850 (*p* = 0.032, *p* < 0.05) between ABTS_IC50 and PCA revealed a strong inverse correlation with a negative Pearson coefficient. According to this result, plant antioxidant activity seems to have been highly influenced by the number and position of the hydrogen-donating hydroxyl groups. 

Also, a remarkable strong correlation between TP and DPPH_IC50 was detected with statistical significance (r = −0.895, *p* = 0.016), which confirms the real interrelation that exists between the data.

As expected, the flavones also had a major contribution to amplifying the antioxidant effect, a phenomenon explained by the strong correlation observed between the concentration of flavones and the IC 50 values determined by all three methods (FL vs. ABTS_IC50: r = −0.774, *p* = 0.071; FL vs. DPPH_IC50: r = −0.841, *p* = 0.036; FL vs. FRAP_EC50: r = −0.841, *p* = 0.036).

Furthermore, the concentration of phenolcarboxylic acids and DPPH_IC50 are perfectly correlated (r = −0.951, −0.9 > r > −1), having a highly statistically significant *p*-value (*p* = 0.004, *p* < 0.01).

Nevertheless, a moderate correlation between TP and the antioxidant effect expressed as FRAP_EC50 was revealed (r = −0.474, *p* = 0.342), a result similar to the one obtained between PCA and FRAP_EC50 (r = −0.626, *p* = 0.184), even if statistical significance was not achieved, possibly due to the much lower sensitivity of the FRAP method.

Regarding the relationship between the secondary metabolites, our results suggest a strong correlation between them (PCA vs. TP: r = 0.893, *p* = 0.016; PCA vs. FL: r = 0.732, *p* = 0.098; TP vs. FL: r = 0.733, *p* = 0.098), these chemical compounds being considered the basic building blocks with essential roles in plant life (resistance, environmental adaptation, and growth) and with important biological significance.

Additionally, it can be seen that there is a statistically significant, perfectly well-correlated relationship between the two most sensitive antioxidant techniques (DPPH vs. ABTS: r = 0.961, *p* = 0.002) and a moderate correlation between FRAP and other antioxidant methods (FRAP vs. ABTS: r = 0.474, *p* = 0.342; FRAP vs. DPPH: r = 0.640, *p* = 0.171).

The positive or negative sign of the Pearson coefficient calculated for pairs of experimental data underlines the direction of the compared variables, i.e., a positive correlation signifies a relationship of direct proportionality, and a negative correlation indicates inverse proportionality between the antioxidant effect of plant extracts (IC50 or EC50 values) and the concentration of chemical compounds.

In order to highlight much more easily and clearly how closely related our analyzed variables are, we created the Heatmap Correlation Matrix (HCM). 

A heatmap correlation is a graphical representation in which Pearson correlation coefficients are displayed using a color-coded matrix. Using this design method, we wanted to outline a complete and very suggestive, but simplified, picture of the correlations established between the antioxidant capacity given by plant extracts and the concentration of active phytoconstituents.

**Figure 1:**
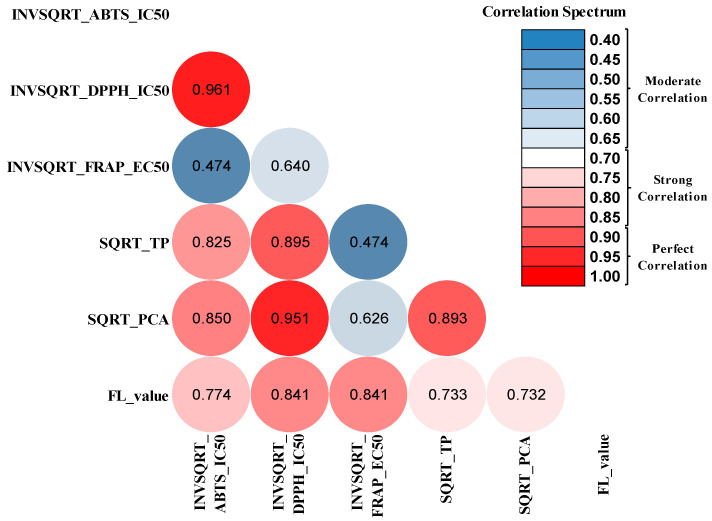
Heatmap correlation matrix and correlation spectrum (moderate correlation: [0.40–0.69]; strong correlation: [0.70–0.89]; perfect correlation: [0.90–1.00];│r│ = absolute value of Pearson correlation coefficient; INVSQRT = inverse square root transformation of data; SQRT = square root transformation of data).

For every association, each cell was color-coded adequately (three-color scale) using specific rules to represent the strength of each correlation. The implementation of the matrix in Figure 1 started with premade correlation plots created in SPSS and was then customized accordingly.

Therefore, in Figure 1, after applying the matrix setting, we can observe that any cell with a correlation coefficient value of 0.4 would be colored blue, the cells with a value of 0.7 would be colored white, and the cells with a value of 1 would be colored red. A color gradient will be obtained in the range of values [0.4–1], whose shades will indicate the strength of correlation, which will make the matrix much easier for the reader to understand. In the range of values, the minimum point is 0.4, the midpoint is 0.7, and the maximum point is +1. Any value between these points would have the shade of color that represents that correlation coefficient value.

We can easily see in the HCM presented in Figure 1 that the correlations between DPPH_IC50 vs. ABTS_IC50 (│r│ = 0.961) and between PCA vs. DPPH_IC50 (│r│ = 0.951) are the perfect correlations (very strong correlations) because they are the cells with the darkest shade of red.

Also, the correlations between TP vs. FRAP_EC50 (│r│ = 0.474) and FRAP vs. ABTS (│r│ = 0.474) turned out to be moderate correlations colored in the darkest shade of blue.

As the color gradient scale moves from dark blue, light blue to white, pale pink, pink to light red, bright red, and deep red, the Pearson correlation coefficients follow each other, corresponding to the various intermediate shades, revealing and quantifying the relationship spectrum.

When constructing the heatmap correlation, the absolute values (│r│) for the Pearson correlation coefficient were taken into account in order to visualize more clearly the magnitude of the association and strength of the correlations without signs and directions.

#### 3.3.3. Evaluation of the Antioxidant Behavior Pattern of Plant Extracts

The mirror pyramids model was the basis for the analysis of the defining antioxidant behavior pattern for each plant extract studied. 

Thus, the plant extracts were grouped according to the similarity of the pyramid shape, and, following the comparison, they could be classified as potent or less potent antioxidants.

The evaluation was carried out based on the values of the antioxidant inhibition of the free radical activity induced by the plant extracts for the sensitive methods, ABTS and DPPH.

**Figure 2:**
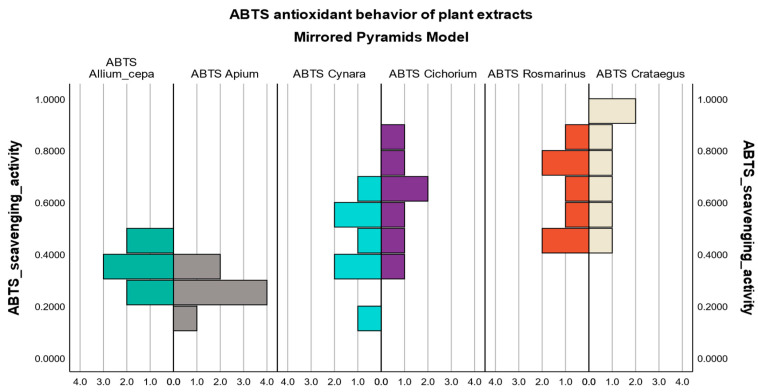
Pyramid model for the ABTS method.

**Figure 3:**
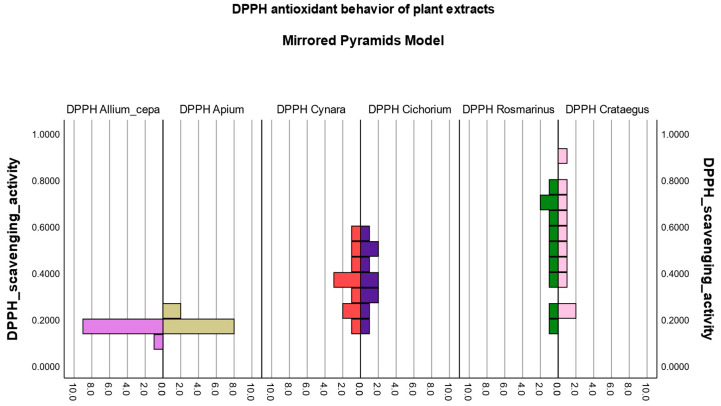
Pyramid model for the DPPH method.

In the case of the ABTS and DPPH methods, it can be seen from Figure 2 and Figure 3 that the pyramid graphs show a high degree of symmetry for some of the analyzed extracts.

It is highlighted that onion extract and celery extract have a similar antioxidant profile, both having the same common method of extraction and preparation from fresh vegetal products.

The artichoke extract and the chicory extract illustrate common and comparable antioxidant behavior in the ABTS and DPPH pyramid models, while the rosemary extract and the hawthorn extract present a very potent antioxidant profile, both registering high values of inhibition of free radicals tested, which suggests their remarkable antioxidant power to annihilate reactive species inducing oxidative stress.

In the antioxidant inhibition boxplot (Supplementary Materials Figures S6 and S7), we can also identify the distribution of the scavenging activity for each vegetal extract in the same outcome area, confirming the hypothesis of pyramid models. 

**Figure 4:**
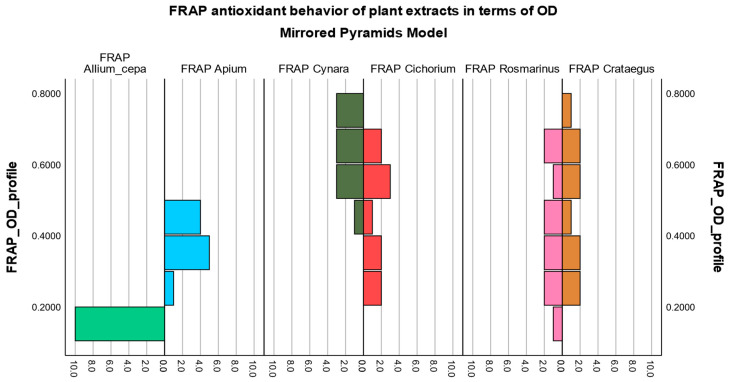
Pyramid model for the FRAP method.

For the FRAP method, the pyramid model and boxplot diagram were created using the values of optical density (Figure 4 and Supplementary Materials Figure S8) because they are proportional to the scavenging activity induced over the ferric ions, and in this way, the antioxidant behavior will be comparable with the ones obtained with the other two methods that have non-physiological radicals.

The same grouping of vegetal extracts remains valid according to similarities (*Allium_cepa* vs. *Apium*, *Cynara* vs. *Cichorium*, *Rosmarinus* vs. *Crataegus*), but visible asymmetries can be noticed regarding the first group made up of onion and celery. These asymmetries may be due to the instability of the active chemical compounds in the fresh vegetal product together with the low sensitivity of the FRAP method. 

The other groups showed the same antioxidant behavior in terms of optical density, as illustrated in the case of inhibition against DPPH or ABTS, with rosemary extract and hawthorn extract reporting the best antioxidant values by all three antioxidant methods.

Looking at Table 3, the antioxidant behavior pattern of plant extracts is also confirmed by the IC50 values obtained for each vegetal extract according to the respective method. 

Rosemary extract has demonstrated the highest antioxidant activity by the DPPH method (RSE: IC50_DPPH_ = 0.11 mg/mL) together with *Crataegi extractum* (CGE: IC50_DPPH_ = 0.11 mg/mL), having the lowest IC50 value, which outlines the potent antioxidant profile of these extracts. Moreover, for the ABTS method, CGE and RSE were also the most effective radical scavengers of the ABTS radical (CGE: IC50_ABTS_ = 0.03 mg/mL; RSE: IC50_ABTS_ = 0.04 mg/mL).

The ferric ion-reducing antioxidant capacity was much better for the artichoke extract, with CE recording the lowest EC50 value by this method (CE: EC50_FRAP_ = 0.06 mg/mL). However, the following extracts that showed a high antioxidant effect were also CGE (EC50_FRAP_ = 0.13 mg/mL) and RSE (EC50_FRAP_ = 0.15 mg/mL). 

The ability to scavenge free radicals by RSE and CGE evaluated by all three antioxidant techniques was very close to the IC50 value of the reference (IC50 ascorbic acid = 0.0165 mg/mL). 

The lowest antioxidant effect reported in our study was available for celery extract, having the highest IC50 value of all six extracts, regardless of the type of method (AGE: IC50_ABTS_ = 2.66 mg/mL; IC50_DPPH_ = 10.31 mg/mL; EC50_FRAP_ = 2.65 mg/mL). In accordance with the pyramid behavior model, onion extract was the modest antioxidant agent, taking into account the results of the DPPH and FRAP methods (ACE: IC50_DPPH_ = 1.32 mg/mL; EC50_FRAP_ = 1.41 mg/mL).

### 3.4. Computational Studies

Based on the UHPLC-MS assay, we selected rosmarinic acid (RA) and chlorogenic acid (CGA) for further in silico studies in order to predict potential pharmacological mechanisms involved in hypolipidemic and anti-obesity activities. Predictions using the PASS algorithm revealed probabilities higher than 50% for RA to exert antioxidant, free radical scavenger, lipid peroxidase inhibitor, hypolipidemic, cholesterol antagonist, and peroxisome proliferator-activated receptor agonist activities, while for CGA the algorithm predicted antioxidant, reductant, free radical scavenger, lipid peroxidase inhibitor, hypolipidemic, and lipid metabolism regulator activities (Supplementary Materials Table S18). Moreover, SwissTargetPrediction and SEA approaches predicted for both phytochemical inhibitory activities on several carbonic anhydrase subtypes. Most notably, both compounds were predicted as inhibitors of carbonic anhydrase 5A and 5B isoforms (Supplementary Materials Table S19), both being expressed at the mitochondrial level. Interestingly, carbonic anhydrase 5A (CA5A) was shown to be involved in the hepatic synthesis of fatty acids and to be a promising therapeutic target for obesity management [91]. Therefore, we chose CA5A as a putative molecular target for RA and CGA. Acetazolamide, a non-selective CA inhibitor, was also used as a positive control for both predictions. The positive control had a 0.2915 probability to inhibit CA5A and a maximum Tanimoto coefficient of 1 for the same target, highlighting that SEA prediction is more reliable on identifying true inhibitors in this case.

Furthermore, we used the YASARA Structure to build homology models of human CA5A, considering that only the mouse homologue was previously crystallized. Using two mouse CA5A structures as templates (1KEQ and 1DMY), we built two models with different conformations of the binding site, one with a catalytic water bound to Zn^2+^ (1KEQ), and another with acetazolamide (a nonselective carbonic anhydrase inhibitor) directly bound to the catalytic Zn^2+^ (1DMY). We chose two models for molecular docking considering that the selected phytochemicals would either potentially bind the water molecule through the phenolic hydroxyl groups or interact through attractive charges via the carboxyl moiety. The quality of the predicted structures was assessed with MolProbity, both models showing good scores for all the evaluation metrics, as shown in Supplementary Materials Table S20. Both models had exceptionally good clash scores, falling within the 100th percentile. Only three side chains had conformations that are statistically unfavorable in the 1KEQ model, while only one poor rotamer was observed for the 1DMY model. Moreover, the 1KEQ model had 96.35% favored rotamers, while the 1DMY model showed 97.72% favored side chain conformations. The Z-score of the 1KEQ model was negative, while the score of the 1DMY model had a value very close to 0, suggesting that both structures had quality scores close to the mean of high-quality reference structures. Furthermore, none of the predicted structures had deviations of Cβ atom positions higher than 0.25 Å, while a very small proportion of bond lengths and angles were outside ideal ranges. Ramachandran plot analysis revealed that no residues were modeled as Ramachandran outliers, while 97.63% of residues were considered as Ramachandran favored for the model based on 1KEQ, and 98.72% were favored for the model based on 1DMY (Supplementary Materials Figure S9). The 1KEQ model had a MolProbity score closer to 1, highlighting the higher quality of this particular model.

Molecular docking simulations were carried out using the validated 3D structures of human CA5A. Caffeic acid (CFA) and acetazolamide (AZM) were docked as positive controls for comparison. We chose CFA as a positive control for the homology model, which included the zinc-bound water molecule, considering the following two factors: it was previously experimentally validated as a CA5A inhibitor (K_i_ = 6.49 µM) [79], and it was also identified in four out of the six assessed plant extracts. CFA showed a binding energy of −5.820 kcal/mol and had a docked pose in the active site, which reflects the expected binding behavior, engaging in hydrogen bonding with the catalytic water molecule and relevant residues such as Thr236, Gln103, and Gln128. Hydrophobic interactions were also formed with residues His130, Val157, and Leu234 within the binding pocket. AZM was docked in the active site of the water-free structure and had a predicted binding energy of −7.140 kcal/mol. Unsurprisingly, the sulfonamide moiety formed a metal bond with the catalytic zinc and hydrogen bonds with Glu142 and Thr235. Moreover, the 1,3,4-thiadiazole ring engaged in pi-alkyl interactions with Leu234 and pi-sulfur and pi-pi T-shaped interactions with His130 (Supplementary Materials Figure S10).

**Figure 5:**
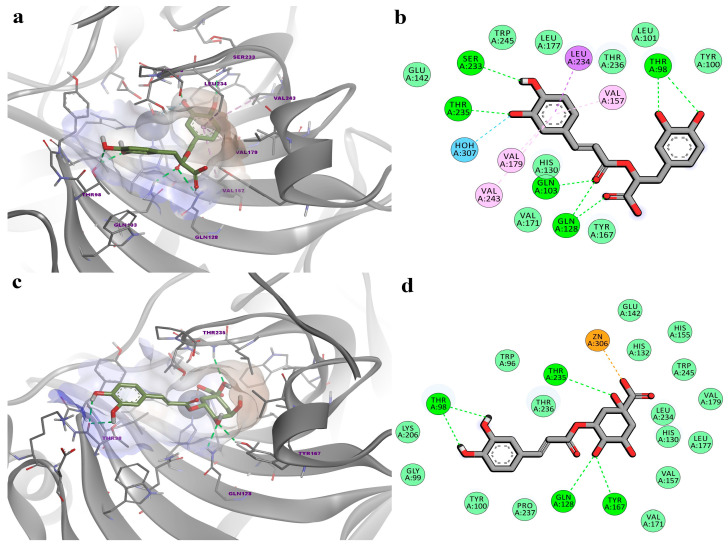
Predicted binding poses of RA and CGA in CA5A active site. (**a**) Predicted conformation of RA-CA5A complex; (**b**) 2D diagram of predicted interactions between RA and CA5A; (**c**) predicted conformation of CGA-CA5A complex; (**d**) 2D diagram of predicted interactions between CGA and CA5A. Green dashes—hydrogen bonds, blue dashes—hydrogen bond with water molecules, orange dashes—attractive charges, purple dashes—pi-sigma interactions, pink dashes—pi-alkyl interactions, light green circles—van der Waals interactions.

Docking studies showed that RA has a higher potential to inhibit CA5A activity by interacting with the catalytic water bound to the Zn atom (1KEQ template), while CGA was more likely to favorably interact with the inhibitor-bound active site conformation of CA5A by directly binding the catalytic Zn^2+^, similar to acetazolamide (1DMY template). The different behavior of the two ligands is particularly interesting, considering that both phytochemicals are condensation products of the known CA5A inhibitor caffeic acid. Docking calculations yielded a binding energy of −7.235 kcal/mol for RA and −7.465 kcal/mol for CGA, both values being lower than those obtained for the positive controls. As observed in Figure 5a,b, similar to positive control CFA, RA formed a hydrogen bond with the zinc-bound water molecule and four more hydrogen bonds with Thr98, Ser233, and Thr235 through the phenolic hydroxyl groups. Moreover, the carboxyl group engaged in hydrogen bonding with Gln128, while the ketone oxygen formed a hydrogen bond with Gln103. The protein–ligand complex is further stabilized by hydrophobic interactions, such as pi-sigma interactions with Leu234 and pi-alkyl interactions with three valines. On the other hand, CGA directly interacted with the catalytic Zn^2+^ through the carboxyl group via attractive charges, while the hydroxyl group from the α-carbon formed a hydrogen bond with Thr235 (similar to AZM). The phenolic hydroxyls also formed hydrogen bonds with Thr98, Gln128 and Tyr167 (Figure 5c,d). Notably, Thr235 is a conserved residue among CA subtypes that is believed to be involved in the binding of phenolic inhibitors [78].

**Figure 6:**
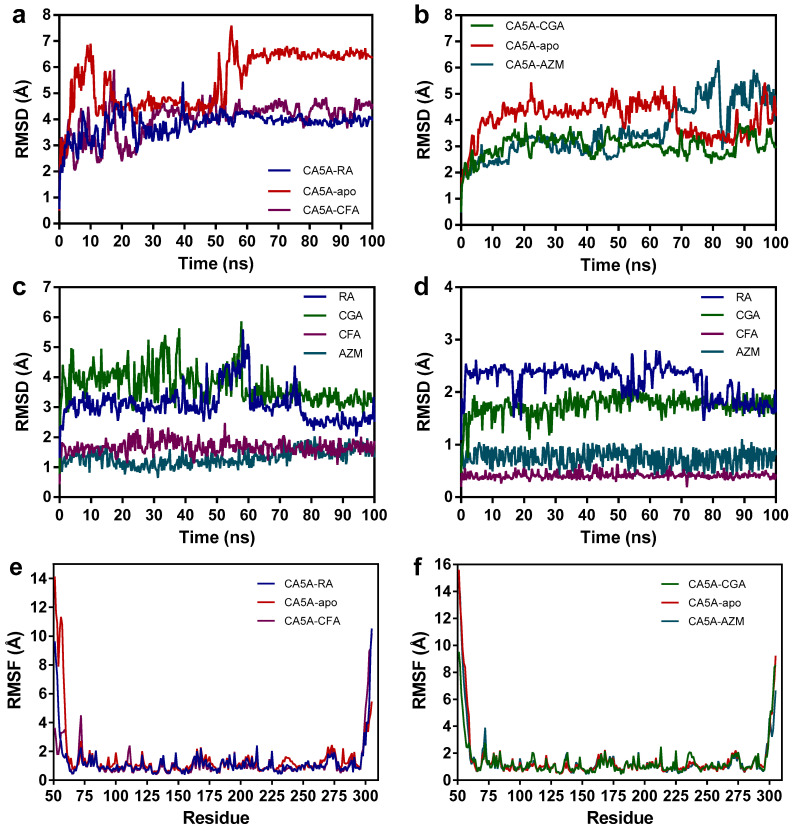
MD results after 100 ns of simulation time. (**a**) RMSD values for all protein carbon atoms as a function of simulation time for CA5A-RA vs. control; (**b**) RMSD values for all protein carbon atoms as a function of simulation time for CA5A-CGA vs. control; (**c**) Ligand movement RMSD after superposing on the receptor for RA and CGA, illustrating the movement of the ligand in the binding pocket; (**d**) Ligand conformation RMSD after superposing on the initial ligand coordinates, illustrating the conformational changes of the ligand; (**e**) RMSF values per amino acid residue for CA5A-RA complex vs. control; (**f**) RMSF values per amino acid residue for CA5A-CGA complex vs. control.

The two predicted protein–ligand complexes were thereafter subjected to molecular dynamics (MD) simulations for 100 ns, using the simulation of the ligand-free structures as controls. The same positive controls were also used for the MD experiments. According to the root mean square deviation (RMSD) of protein carbon atoms as a function of time, we noted that the CA5A-RA complex reached a relative equilibrium after 25 ns, while the equilibration period lasted approximately 15 ns for the CA5A-CGA complex (Figure 6a,b). Therefore, further analysis was performed for the last 75 ns for the CA5A-RA complex and the last 85 ns for the CA5A-CGA complex. In both cases, MD results showed that the protein–ligand complexes are significantly more stable than the apo structure of CA5A (3.919 Å vs. 5.776 Å average RMSD for CA5A-RA, 3.093 Å vs. 4.169 Å average RMSD for CA5A-CGA), indicating a high probability for the two phytochemicals to bind to the active site of the target protein. The positive control CA5A-CFA complex had a similar behavior to CA5A-RA complex (4.269 Å average RMSD), while the CA5A-AZM complex (3.810 Å average RMSD) behaved similarly to CA5A-CGA only in the first 65 ns simulation time, the latter being overall more stable. Furthermore, CGA showed higher movement within the binding site (Figure 6c), while RA had a higher conformational change during the simulation time (Figure 6d). Both positive controls showed lower ligand conformation RMSD and ligand movement RMSD values, probably due to their significantly smaller structures. The CA5A-RA complex had higher mean numbers of intramolecular hydrogen bonds than the apo structure (186.1 vs. 176.5), while the ligand-free structure formed more intramolecular hydrogen bonds than the CA5A-CGA complex (173.9 vs. 180.3, Supplementary Materials Figure S11a,b). Similar to the positive control complexes, both CA5A-RA and CA5-CGA complexes were more compact than the apo structures, considering that radius of gyration (Rg) values were overall lower during the simulation time (Supplementary Materials Figure S11c,d). The root mean square fluctuation values for each amino acid residue are shown in Figure 6e,f. As expected, lower RMSF values were obtained for the residues involved in interactions with the two ligands when compared to the negative control simulation.

**Figure 7:**
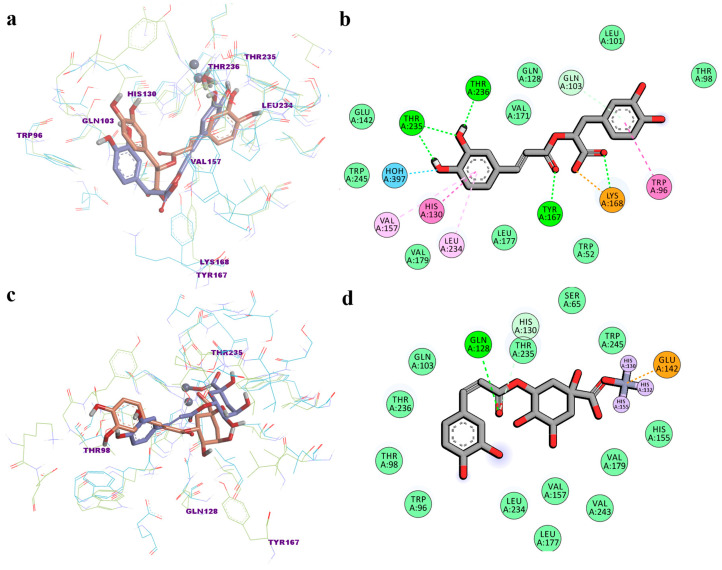
Ligand binding pose after 100 ns of simulation. (**a**) Superposition of the final snapshot of CA5A-RA complex simulation (blue/purple) on the initial conformation (green/orange); (**b**) 2D diagram of molecular interactions between RA and CA5A after 100 ns; (**c**) Superposition of the final snapshot of CA5A-CGA complex simulation (blue/purple) on the initial conformation (green/orange); (**d**) 2D diagram of molecular interactions between CGA and CA5A after 100 ns. Green dashes—hydrogen bonds, blue dashes—hydrogen bond with water molecules, orange dashes—attractive charges, magenta dashes—pi-pi T-shaped interactions, pink dashes—pi-alkyl interactions, tea green dashes—carbon–hydrogen bonds, light green circles—van der Waals interactions.

For the analyzed trajectories, RA showed free energy of binding values between −91.012 and −20.296 kcal/mol (−50.486 ± 11.543 kcal/mol), and −132.834 and −20.912 kcal/mol (−64.873 ± 18.815 kcal/mol) for CGA, which were calculated using the MM/PBSA approach. Comparably, the free binding energies of positive controls ranged between −126.234 and −31.051 kcal/mol (−81.706 ± 12.583 kcal/mol) for CFA and between −165.592 and −17.114 kcal/mol (−77.114 ± 16.795 kcal/mol) for AZM. We further chose to discuss the protein–ligand interactions noted after the 100 ns simulation. The superposition of the final snapshot conformation of the two complexes on the initial conformations is illustrated in Figure 7a,c. Notably, in the case of RA, the hydrogen bond with the zinc-bound water molecule was stable over the 100 ns simulation, while two pi-pi T-shaped interactions appeared between the two phenol moieties and His130 and Trp96, respectively, which could suggest a higher stability of the complex. Moreover, the positively charged residue Lys168 rotated during the simulation time to form a hydrogen bond and attractive charge interactions with the carboxyl moiety of RA (Figure 7b). On the other hand, CGA rotated its carboxyl moiety and migrated closer to the catalytic zinc, forming a coordinate covalent bond with the metal atom. Furthermore, polar interactions were observed between the ketone oxygen and Gln128 and His130, respectively (Figure 7d). The free binding energy was −58.579 kcal/mol for RA and −61.268 kcal/mol for CGA after 100 ns. 

## 4. Discussion

Plant metabolites are essential compounds that can exhibit several noticeable effects in the human body due to their structural biosimilarity. Phenolic compounds are a very important class of phytocompounds with varying significance, being responsible for multiple beneficial actions at the cellular and tissue levels that have been demonstrated in previous studies [71,92].

The antioxidant capacity is attributed to a combination of polyphenols because of their extraordinary ability to protect the body from oxidative damage. These are the chief components of plant extracts, which are considered of major relevance in the total antioxidant activity of the living systems for a lot of medicinal plants, acting as free radical scavengers [60,93,94]. 

Overproduction of reactive species in the living organism is an important trigger of oxidative stress, which is associated with the oxidative destruction of functional cells, nucleic acids, enzymes, or proteins. Simultaneously, this destruction may lead to severe chronic degenerative or metabolic diseases, also affecting the synthesis, accumulation, and redistribution of lipids at the tissue level [4]. 

New insights were generated through our results, the novelty of the study being the analysis of the extracts obtained from fresh vegetal sources (*Allii cepae extractum* and *Apii graveolentis extractum*) together with extracts obtained from dry vegetal material (*Rosmarini extractum*, *Cichorii extractum*, *Cynarae extractum*, and *Crataegi extractum*). In order to explain and confirm their potential therapeutic benefits for ameliorating dysregulated blood lipid profiles, we could mix them in future studies and test their synergistic effects. Also, onion green aerial parts have not been intensively researched; the available studies regarding onion extract refer to the underground herbal product [30,95].

Even if the plant products were purchased as single-component teas or as plant products in dried or fresh form, from the local pharmaceutical market or from the food chain, we made sure from the beginning of the study that the producers are Romanian and the species are indigenous.

Therefore, although they are well-studied medicinal species, pedoclimatic conditions in our country can influence the biosynthesis of active ingredients and also their concentrations, antioxidant profiles, or therapeutic potency. We consider that this study can open new perspectives in terms of the characterization of medicinal plants/plant products that are used by indigenous producers in obtaining dietary supplements, providing possible information on potential doses in administration, as well as the period of administration.

The current research results reveal the outstanding antioxidant profile of six plant extracts that could be used as potent metabolic agents in the treatment or prevention of hyperlipemias of various causes.

Among the polyphenolic compounds, those that contribute more to the manifestation of the antioxidant activity of plant extracts are mainly total polyphenols, phenolic acids, and flavones [96]. 

According to our spectrophotometric outcomes, the highest significant concentrations of active chemical compounds relevant for exerting the antioxidant effect were recorded for rosemary extract (39.62 ± 13.16 g total polyphenols/100 g DE expressed as tannic acid; 22.05 ± 1.31 g phenolic acids/100 g DE expressed as chlorogenic acid) and hawthorn extract (5.32 ± 0.26 g flavones/100 g DE expressed as rutoside; 25.93 ± 1.10 g total polyphenols/100 g DE expressed as tannic acid; 14.05 ± 1.65 g phenolic acids/100 g DE expressed as chlorogenic acid). However, significant concentrations were also observed for the rest of the studied extracts, which outlines the special phytochemical profile with possible therapeutic implications. In the case of artichoke extract and chicory extract, high concentrations of TP and PCA compared to the concentration of flavones were highlighted, which can justify the proven antioxidant capacity in vitro.

After we performed the UHPLC-MS method, it could be observed that chlorogenic acid and rosmarinic acid showed particularly high concentrations, indicating their abundance in the respective plant extracts. The presence and quantity of these compounds contribute significantly to the antioxidant and potential health benefits of the extracts.

Regarding the low polyphenol concentrations recorded by onion extract using the UHPLC-MS method, we can highlight the factors that could influence the phytochemical composition. Scallions, or green onions, are commonly used for their aromatic properties in culinary applications, and while they contain beneficial compounds, they may not accumulate polyphenols to the same extent as other herbs or leaves with higher chlorophyll content or known for their rich polyphenolic profiles, such as rosemary or artichoke leaves. Factors that could contribute to lower polyphenol concentrations in scallions may include the following:

Plant Variety and Part Used: Different parts of the plant can have varying concentrations of polyphenols. For example, bulb onions are known to have higher phenolic content compared to the green parts [97].

Cultivation Conditions: Soil composition, sunlight exposure, and water availability can affect polyphenol biosynthesis in plants. Green onions, often grown for their quick yield and not necessarily for maximum polyphenol content, may have lower levels due to these cultivation practices [98].

Harvesting Time: The stage of growth at which plants are harvested can significantly influence their polyphenol content. It is possible that the scallions were harvested at a time when polyphenol levels were not at their peak [99].

Post-Harvest Handling: Exposure to light, temperature changes, and processing can lead to the degradation of polyphenols. If scallions are not handled or processed optimally post-harvest, this could lead to lower polyphenol content [100].

Extraction Method: The efficiency of the used extraction method can also impact the measured polyphenol content. If the extraction conditions were not optimized for the polyphenols present in scallions, this might result in lower extraction yields [98].

Given these considerations, the modest concentration of polyphenols in the onion extract sample could be attributed to a combination of these factors rather than a single cause. It is important to optimize each stage from cultivation to extraction to ensure the maximum yield of polyphenolic compounds for analysis.

The low concentrations of gallic acid, abscisic acid, ellagic acid, and *p*-coumaric acid in all of the analyzed samples, after quantification by the UHPLC-MS method, can be attributed to several factors intrinsic to the nature of these compounds and the characteristics of the plant materials from which they were extracted.

- Gallic Acid: Known for its potent antioxidant properties, gallic acid’s concentration in plant extracts can be influenced by the plant’s metabolism, growing conditions, and the specific plant part being analyzed. Its lower detection could be due to the preferential biosynthesis of other phenolics in the plant [101].
- Abscisic Acid: Primarily recognized for its role as a plant hormone regulating growth and development, abscisic acid’s presence in lower concentrations might reflect its specific regulatory functions rather than its accumulation as a secondary metabolite like other polyphenols. Its detection levels can also be affected by the extraction and analysis methods, which may not be optimized for hormone detection [102].
- Ellagic Acid: This compound is derived from the hydrolysis of ellagitannins, and its lower concentration could be indicative of the levels of these precursor compounds in the plants analyzed. The variation in ellagic acid content can be significantly influenced by genetic factors, environmental conditions, and the maturity of the plant material at the time of harvest [103].
- *p*-Coumaric Acid: As a phenolic acid, *p*-coumaric acid’s lower concentrations might be due to its role as a building block in the biosynthesis of more complex polyphenols, such as flavonoids and lignins, rather than as an end-product accumulated in the plant tissues. Environmental factors and plant stress can also modulate its biosynthesis [104].

Recent studies have underscored the complexity of polyphenol biosynthesis, metabolism, and accumulation in plants, highlighting the impact of genetic, environmental, and methodological factors on the quantification of these compounds in plant extracts. The lower concentrations observed for these specific polyphenols across the analyzed samples underscore the need for targeted approaches in the extraction and analysis to accurately quantify these and other bioactive compounds.

Vanillic and ferulic acids, identified in the analyzed samples, play significant roles due to their biochemical properties. These compounds are widely recognized for their antioxidant activities, contributing to the protection against oxidative stress by scavenging free radicals and chelating metal ions. Additionally, they exhibit potential hypolipidemic effects, which could be beneficial in the management and prevention of cardiovascular diseases. Vanillic acid has also been studied for its anti-inflammatory and antimicrobial activities, indicating its broad spectrum of potential health benefits [105].

Ferulic acid was detected by the UHPLC-MS method in all analyzed extracts; the highest values were found for chicory and rosemary extracts. Ferulic acid’s role extends beyond its antioxidant activity, as it has been shown to exhibit anti-inflammatory, anticancer, and antidiabetic effects, contributing to its importance in disease prevention and management [106].

In the context of the specific analyzed samples, vanillic and ferulic acids contribute to the overall antioxidant profile of the plant extracts. Their presence, even in low concentrations, enhances the extracts’ capacity to mitigate oxidative stress and potentially lowers the risk of chronic diseases associated with oxidative damage and inflammation. The polyphenolic content of these samples, including vanillic and ferulic acids, underscores the health-promoting potential of the plants from which they were derived.

Given that all of the target compounds were well separated under the described UHPLC-MS conditions, some limitations of the method still remain important to consider. The method exclusively uses a 70% methanol solution for extraction, which, although effective for the compounds of interest in this study, may not extract other phytochemicals efficiently. This could lead to an incomplete characterization of the plant extracts’ chemical profiles if additional compounds are of interest in future studies. The use of a relatively short column (50 mm) and a fast flow rate (0.8 mL/min) provided adequate separation for this specific set of compounds, but this setup might limit the resolution in analyses requiring separation of a broader range of compounds with more subtle differences in polarity or molecular structure. Additionally, operating exclusively in negative ion mode may preclude the detection of compounds that ionize more efficiently in positive ion mode, which could be relevant for comprehensive plant profiling or when extending the method to other types of samples. Lastly, the fixed environmental conditions—column temperature at 28 °C and sample storage at 10 °C—although suitable for this analysis, may not be optimal for all analytes, particularly those sensitive to temperature fluctuations. These aspects should be considered in method development when adapting to other analytical goals or different sample types.

The present study showed strong antioxidant activity for rosemary extract (IC50_ABTS_ = 0.04 mg/mL; IC50_DPPH_ = 0.11 mg/mL; EC50_FRAP_ = 0.15 mg/mL) and hawthorn extract (IC50_ABTS_ = 0.03 mg/mL; IC50_DPPH_ = 0.11 mg/mL; EC50_FRAP_ = 0.13 mg/mL), with a statistically significant difference compared to the other plant extracts (*p* < 0.05). The variation of the scavenging effect between the two extracts is practically imperceptible in vitro, both RSE and CGE being potent antioxidants (a lower IC50 value signifies higher antioxidant activity). Although, in the case of the FRAP method, the artichoke extract was the one that presented the highest antioxidant activity, reaching the lowest EC50 value for this technique (EC50_FRAP_ = 0.06 mg/mL). This unexpected result was presumably due to the greater stability of chemical compounds in the extraction solvent (50% ethanol), which can contribute better to the total antioxidant capacity, as mentioned in previous research [107]. The highest IC50 values were detected for celery extract; these findings clearly confirm the lowest free radical scavenging capacity of this extract, which is attributed to the lowest concentrations of polyphenolic compounds.

Overall, the Pearson assay and the heatmap correlation matrix indicate that the characterized chemical compounds in our work for each analyzed extract were correlated with their antioxidant effects, as was considered and demonstrated in many other studies. The extracts revealed a good correlation spectrum with significant values for Pearson correlation coefficients.

The antioxidant behavior pattern of plant extracts was evaluated through pyramid models and boxplot diagrams. Significant changes in the antioxidant profile were observed for onion extract and celery extract since they were obtained from fresh vegetal raw material with the lowest extraction yields. At the same time, 70% ethanol used as an extraction solvent can cause instability in the extracted phenolic compounds. Instead, rosemary extract and hawthorn extract illustrated a similar antioxidant behavior, positioning themselves in the upper part of the graphs (pyramid models) or in the rightmost part (boxplot diagrams), which confirms the findings made in our study.

Computational approaches were thereafter exploited to predict potential anti-obesity and hypolipidemic mechanisms for rosmarinic acid and chlorogenic acid, two phytochemicals assessed in high quantities in the investigated extracts. We chose only these phytoconstituents for the computational studies on the aforementioned basis, considering that relatively high plasma concentrations should be reached to elicit their predicted therapeutic activity or to specifically bind to a protein target. Using three web-based tools, we identified several potential pharmacological activities that were in line with the scope of our study, such as antioxidant, hypolipidemic, peroxisome proliferator-activated receptor (PPAR) agonist, and lipid metabolism-regulating activities. Interestingly, rosmarinic acid was previously shown to indeed exert its anti-inflammatory and cardioprotective activities through activation of PPAR-gamma [108]. Moreover, both rosmarinic acid and chlorogenic acid were predicted in our study as potential modulators of carbonic anhydrase 5 (CA5) isoforms. CA5 is the only carbonic anhydrase expressed at the mitochondrial level and is involved in endogenous fatty acid biosynthesis and lipogenesis. The two isoforms of CA5, CA5A, and CA5B show different expression patterns, i.e., CA5A is mainly expressed in hepatocytes, whereas CA5B has a larger tissue distribution [91,109]. Carbonic anhydrases are metalloenzymes that catalyze the conversion reactions between CO_2_ and bicarbonate, generating protons and being involved in pH regulation. Due to its involvement in lipid metabolism, CA5A was proposed as a promising therapeutic target for novel anti-obesity pharmacological agents [91]. Previous studies highlighted that several natural polyphenols inhibit enzymatic activities of carbonic anhydrases. For instance, rosmarinic acid was shown to inhibit CA9 and CA12, while both chlorogenic acid and rosmarinic acid inhibited CA1 and CA2 [78,110]. Moreover, caffeic acid and ferulic acid, which were also identified in the investigated plant extracts, were previously shown to inhibit CA5A [79,111]. Therefore, caffeic acid was selected as a positive control for the in silico studies.

Similar to other CA inhibitors [78,112], molecular docking and molecular dynamics simulations revealed that both rosmarinic acid and chlorogenic acid can form stable complexes with the active site of CA5A by either binding the zinc-bound catalytic water molecule or by direct interaction with the catalytic Zn^2+^. The novelty of our extensive computational study is illustrated by the observation that, even though both chlorogenic acid and rosmarinic acid are esterification products between caffeic acid and quinic acid (CGA) or dihydroxyphenyl-lactic acid (RA) and are highly chemically related polyphenols, the two metabolites have completely different in silico behaviors. CGA and RA adopt opposite orientations in the catalytic site of CA5A, i.e., the caffeic acid substructure of RA participated preferably in hydrogen-bonding with the zinc-bound water molecule and the two conserved threonine residues that are usually involved in mediating the interactions between polyphenols and many CA isoforms, while the carboxylic acid moiety formed a salt bridge with a distant lysine; in contrast, the caffeic acid portion of CGA is oriented towards more distant residues, while the carboxylic acid moiety of the quinic acid substructure formed a metal coordinate bond with the catalytic Zn^2+^. Therefore, we herein propose that CGA and RA have the potential to inhibit CA5A by different, opposing binding modalities, which, however, yield protein–ligand complexes with similar stabilities over simulation times.

Our in silico findings suggest that the potential anti-obesity and hypolipidemic activities of *Cynarae* extract, *Crataegi* extract, and *Rosmarini* extract could be partly supported by the inhibition of the hepatic CA5A by rosmarinic acid and chlorogenic acid. Moreover, both compounds were previously shown to possess lipid-lowering effects and to lower body weights and visceral fat mass in several experimental settings, including animal models of obesity, through various molecular mechanisms [113,114,115,116]. 

The following limitations of the in silico study were identified: the length of the MD simulations was not long enough to encompass all the conformational changes that can occur in a physiological medium, considering that significant variations were observed for the positive control CA5A-AZM complex after 65 ns. Moreover, due to the relatively speculative nature of computational studies, in vitro and in vivo assays are warranted to experimentally validate the predicted CA5A inhibitory activity of rosmarinic acid and chlorogenic acid and to thoroughly evaluate the anti-obesity and lipid-lowering activities of the assessed plant extracts. 

Taken altogether, we can suggest that the predicted CA5A inhibitory activity of some of the phytochemicals and the highlighted antioxidant activities can complement each other due to the potentially synergistic action of these phenolic components contained in the studied extracts. Therefore, the antioxidant activity of the plant extracts in conjunction with the potential lipid-lowering activity could provide an effective adjuvant therapeutical option for patients with obesity and dyslipidemia. However, extensive in vitro and in vivo studies, coupled with structure–activity relationship analysis, are needed to further support this hypothesis, using biochemical assays on both CA5 isoforms and animal models of obesity and dyslipidemia. Further exploration of the CA5A inhibitory activity of the phytochemicals that were detected in lower quantities in the prepared extracts by expanding the computational study in corroboration with experimental validation could also add valuable insights into the potential therapeutic application of such plant products.

## 5. Conclusions

The present research focused on the analysis and characterization of the following six plant extracts: *Allii cepae extractum*, *Rosmarini extractum*, *Cichorii extractum*, *Cynarae extractum*, *Apii graveolentis extractum*, and *Crataegi extractum*, to highlight their potential use in dyslipidemia and obesity. Phytochemical analysis revealed that the rosemary extract was the richest in total polyphenols and phenolcarboxylic acids, while the hawthorn extract was rich in flavones. High amounts of chlorogenic acid and rosmarinic acid were detected in both extracts. 

Moreover, the rosemary and hawthorn extracts showed the highest antioxidant activity in vitro, having great medicinal importance due to the presence of high levels of phenolic compounds. However, all the studied extracts were capable of sustaining the idea of their potential use in dyslipidemia and obesity, whether used alone or combined. Computational studies revealed that their major constituents, chlorogenic and rosmarinic acid, have the potential to inhibit mitochondrial carbonic anhydrase 5A and therefore reduce the hepatic biosynthesis of fatty acids. Further studies are warranted to validate the predicted pharmacological mechanism and assess the therapeutic potential of the proposed plant extracts in animal models of dyslipidemia and obesity.

## Figures and Tables

**Table 1:** Quantitative analysis of the main phenolic classes of the analyzed plant extracts.

Plant Extract	FL (g Rutoside/100 g DE)	TP (g Tannic Acid/100 g DE)	PCA (g Chlorogenic Acid/100 g DE)
ACE	2.03 ± 0.12 ^y^	3.77 ± 0.84 ^c^	ND
RSE	3.71 ± 0.18 ^x^	39.62 ± 13.16 ^a^	22.05 ± 1.31 ^o^
CHE	3.64 ± 0.11 ^x^	30.51 ± 1.96 ^a^	6.16 ± 0.79 ^q^
CE	4.38 ± 0.32 ^w^	7.74 ± 0.86 ^b^	4.47 ± 0.27 ^r^
AGE	0.35 ± 0.07 ^z^	0.57 ± 0.06 ^d^	0.38 ± 0.01 ^s^
CGE	5.32 ± 0.26 ^v^	25.93 ± 1.10 ^a^	14.05 ± 1.65 ^p^

Values with different superscript letters are statistically significant at *p* < 0.05 in the column (ANOVA, Games–Howell’s post hoc test). Results are expressed as mean ± SD (*N* = 5). DE: dry extract; ND: not detected; FL: flavones; TP: total polyphenols; PCA: phenolcarboxylic acids.

**Table 2:** Quantification of each polyphenol contents (mg compound/g extract) in each extract using UHPLC–MS.

mg/g Extract	S1	S2	S3	S4	S5	S6
Gallic acid	D	D	Missing	D	D	D
Protocatechuic acid	0.380 ± 0.02	D	D	0.334 ± 0.03	2.165 ± 0.04	0.105 ± 0.01
Chlorogenic acid	78.529 ± 1.15	2.854 ± 0.05	D	6.497 ± 0.42	187.435 ± 1.96	D
Vanillic acid	1.205 ± 0.08	0.748 ± 0.07	1.160 ± 0.05	1.230 ± 0.03	1.117 ± 0.06	1.782 ± 0.07
Caffeic acid	0.173 ± 0.01	D	D	0.968 ± 0.09	0.102 ± 0.01	2.849 ± 0.08
Syringic acid	1.329 ± 0.06	0.783 ± 0.04	1.349 ± 0.03	Missing	1.249 ± 0.04	1.360 ± 0.03
*p*-Coumaric acid	D	D	D	D	0.229 ± 0.01	D
Ferulic acid	0.131 ± 0.01	0.055 ± 0.01	0.211 ± 0.02	0.265 ± 0.02	0.118 ± 0.01	0.242 ± 0.02
Ellagic acid	0.051 ± 0.01	D	D	D	D	D
Rosmarinic acid	0.095 ± 0.01	0.039 ± 0.01	0.045 ± 0.01	0.064 ± 0.02	0.090 ± 0.02	317.100 ± 2.70
Abscisic acid	0.049 ± 0.01	0.015 ± 0.01	D	0.005 ± 0.002	0.062 ± 0.01	D

Legend: S1—*Cynarae* extract (CE), S2—*Apii graveolentis* extract (AGE), S3—*Allii cepae* extract (ACE), S4—*Cichorii* extract (CHE), S5—*Crataegi* extract (CGE), S6—*Rosmarini* extract (RSE), D—detected under the quantification limit.

**Table 3:** Radical scavenging activities expressed as IC50/EC50 antioxidant value.

Plant Extract	IC50 ABTS (mg/mL)	IC50 DPPH (mg/mL)	EC50 FRAP (mg/mL)
ACE	0.18	1.32	1.41
RSE	0.04	0.11	0.15
CHE	0.15	0.34	0.26
CE	0.20	0.37	0.06
AGE	2.66	10.31	2.65
CGE	0.03	0.11	0.13

ABTS: 2,2-azinobis-3-ethylbenzotiazoline-6-sulfonic acid method; DPPH: 2,2-diphenyl-1-picryl-hydrazine method; FRAP: ferric-reducing antioxidant power method.

## Data Availability

The original contributions presented in the study are included in the article/Supplementary Materials, further inquiries can be directed to the corresponding author.

## Supplementary Materials

The following supporting information can be downloaded at: https://www.mdpi.com/article/10.3390/biomedicines12071431/s1, Table S1: Calibration curve characteristics; Table S2: Gradient of mobile phase; Table S3: SMILES codes specific to the compounds used in the computational studies; Table S4: Descriptive statistics for DPPH inhibition results; Table S5: Descriptive statistics for ABTS inhibition results; Table S6: Descriptive statistics for FRAP optical density results; Table S7: ANOVA test for antioxidant DPPH scavenging activity; Table S8: Robust tests (antioxidant DPPH); Table S9: Games–Howell post hoc test for the DPPH method; Table S10: ANOVA test for antioxidant ABTS scavenging activity; Table S11: Robust tests (antioxidant ABTS); Table S12: Games–Howell post hoc test for the ABTS method; Table S13: ANOVA test for antioxidant FRAP scavenging activity; Table S14: Robust tests (antioxidant FRAP); Table S15: Games–Howell post hoc test for the FRAP method; Table S16: The chemical content and antioxidant values of plant extracts; Table S17: Correlations among variables; Table S18: Activity prediction results using PASS algorithm; Table S19: Target prediction using SwissTargetPrediction and SEA; Table S20: Quality parameters for the human CA5A homology models; Figure S1: Comparative graphs of phytochemical screening in plant extracts; Figure S2: The chromatograms obtained using UHPLC–MS method for six extracts; Figure S3: Normal Q-Q plot for DPPH antioxidant activity; Figure S4: Normal Q-Q plot for ABTS antioxidant activity; Figure S5: Normal Q-Q plot for FRAP antioxidant activity; Figure S6: The boxplot distribution of ABTS antioxidant inhibition within groups; Figure S7: The boxplot distribution of DPPH antioxidant inhibition within groups; Figure S8: The boxplot distribution of FRAP optical density within groups; Figure S9: Homology modeling results for human CA5A; Figure S10: Predicted binding poses of positive controls CFA and AZM in CA5A active site; Figure S11: Results following 100 ns MD simulation.
