# Supplementary material for: Phytochemical Characterization and Antioxidant Activity Evaluation for Some Plant Extracts in Conjunction with Pharmacological Mechanism Prediction: Insights into Potential Therapeutic Applications in Dyslipidemia and Obesity
Source: Biomedicines. 2024 Jun 27;12(7):1431. doi: 10.3390/biomedicines12071431 (PMC11274650; doi:10.3390/biomedicines12071431)
Supplement: Supplementary file 1 [file biomedicines-12-01431-s001.zip › biomedicines-3066345-supplementary.pdf]

Article

Phytochemical Characterization and Antioxidant Activity Evaluation for Some Plant Extracts in Conjunction with Pharmacological Mechanism Prediction: Insights into Potential Therapeutic Applications in Dyslipidemia and Obesity

Supplementary Materials

UHPLC-MS Reference preparation

The following reference compounds were used: ellagic acid (Sigma), abscisic acid (Sigma), syringic acid (Merck), ferulic acid (Merck), caffeic acid (Merck), gallic acid (Merck), vanillic acid (Merck), p-coumaric acid (Merck), rosmarinic acid (Sigma), chlorogenic acid (Alfa Aesar) and protocatechuic acid (Merck). 10 mg of each reference compound was solubilized in 10 mL methanol and dilution were made to obtain the calibration curves.

Table S1. Calibration curve characteristics

Compound name	m/z	Retention time [min]	R^2	Fit Type
Gallic acid	169	1.51	0.99805	Quadratic (2nd Order)
Protocatechuic acid	153	2.61	0.99997	Quadratic (2nd Order)
Chlorogenic acid	353	4.03	0.99955	Quadratic (2nd Order)
Vanillic acid	167	4.32	0.99973	Quadratic (2nd Order)
Caffeic acid	179	4.56	0.99887	Quadratic (2nd Order)
Syringic acid	197	4.88	0.99935	Quadratic (2nd Order)
p-Coumaric acid	163	6.60	0.99937	Quadratic (2nd Order)
Ferulic acid	193	7.41	0.99975	Quadratic (2nd Order)
Ellagic acid	301	7.77	0.99991	Quadratic (2nd Order)
Rosmarinic acid	359	9.24	0.99852	Quadratic (2nd Order)
Abscisic acid	263	10.11	0.99990	Quadratic (2nd Order)

Table S2. Gradient of mobile phase

Time [min]	Flow [mL/min]	%A	%B
Initial	0.8	98	2
1.8		91	9
4.0		91	9
10.0		70	30
15.0		10	90
16.0		10	90
17.0		98	2

Computational Studies

Table S3. SMILES codes specific to the compounds used in the computational studies

Compound	Canonical SMILES	Isomeric SMILES
Rosmarinic acid (RA)	C1=CC(=C(C=C1CC(C(=O)O)OC(=O)C=CC2=CC(=C(C=C2)O)O)O)O	C1=CC(=C(C=C1C[C@H](C(=O)O)OC(=O)/C=C/C2=CC(=C(C=C2)O)O)O)O
Chlorogenic acid (CGA)	C1C(C(C(CC1(C(=O)O)O)OC(=O)C=CC2=CC(=C(C=C2)O)O)O)O	C1C(C(C(CC1(C(=O)O)O)OC(=O)C=CC2=CC(=C(C=C2)O)O)O)O
Caffeic acid (CA)	C1=CC(=C(C=C1C=CC(=O)O)O)O	C1=CC(=C(C=C1/C=C/C(=O)O)O)O
Acetazolamide (AZM)	CC(=O)NC1=NN=C(S1)S(=O)(=O)N	-

Results

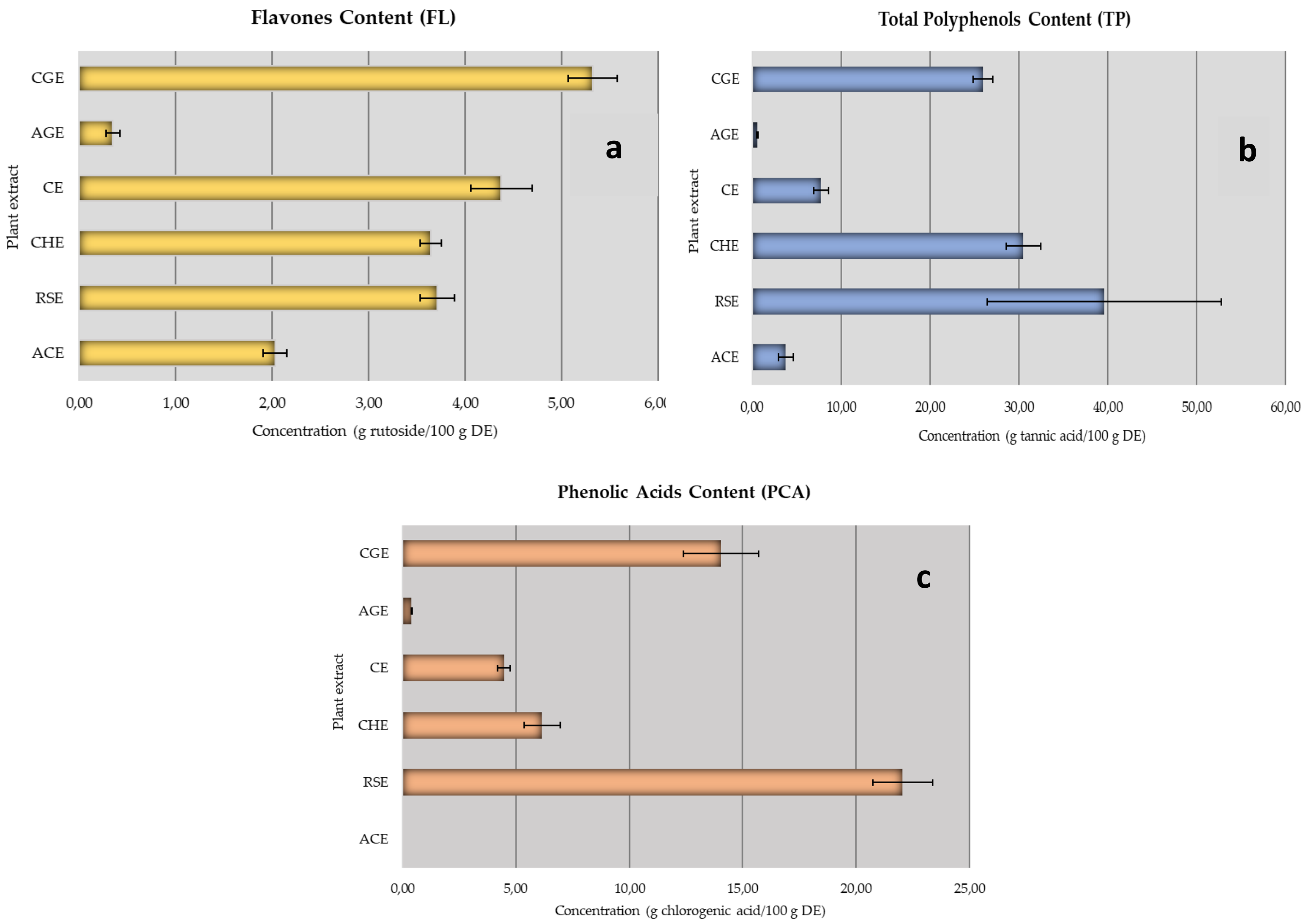

Figure S1. Comparative graphs of phytochemical screening in plant extracts: (**a**) FL by CH_3COONa-$AlCl_3$ assay, (**b**) TP by Folin-Ciocâlteu assay, and (**c**) PCA by Arnow's assay. (*N* = 5, Means with error bars representing SD).

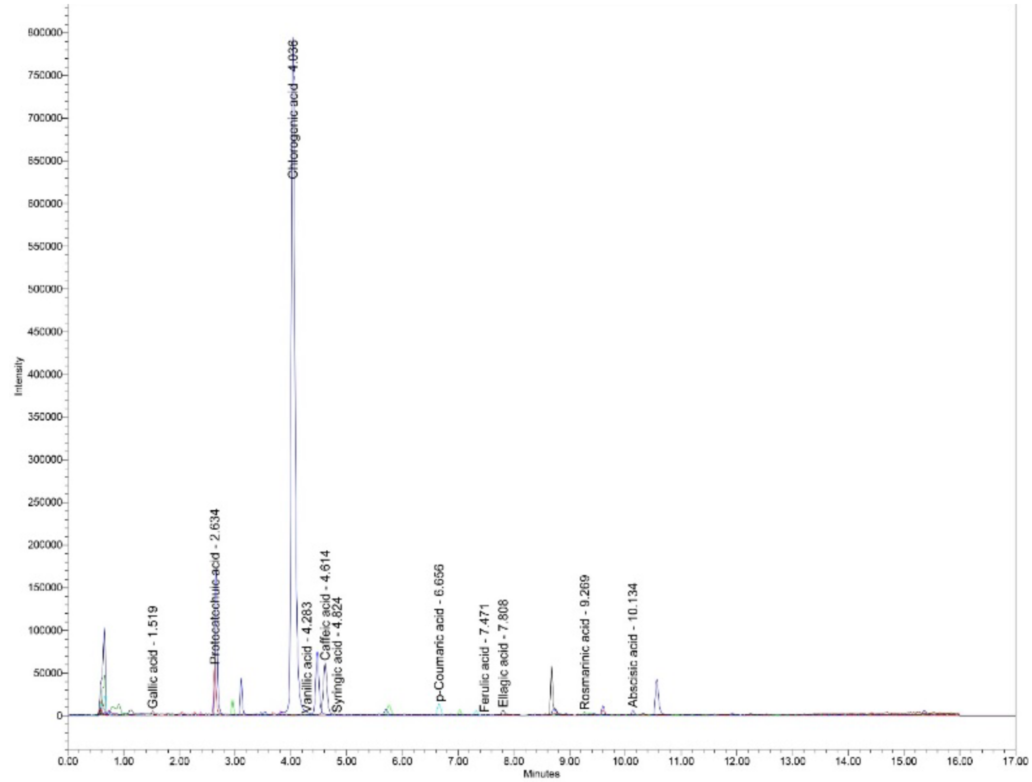

a. *Cynarae* extract – CE

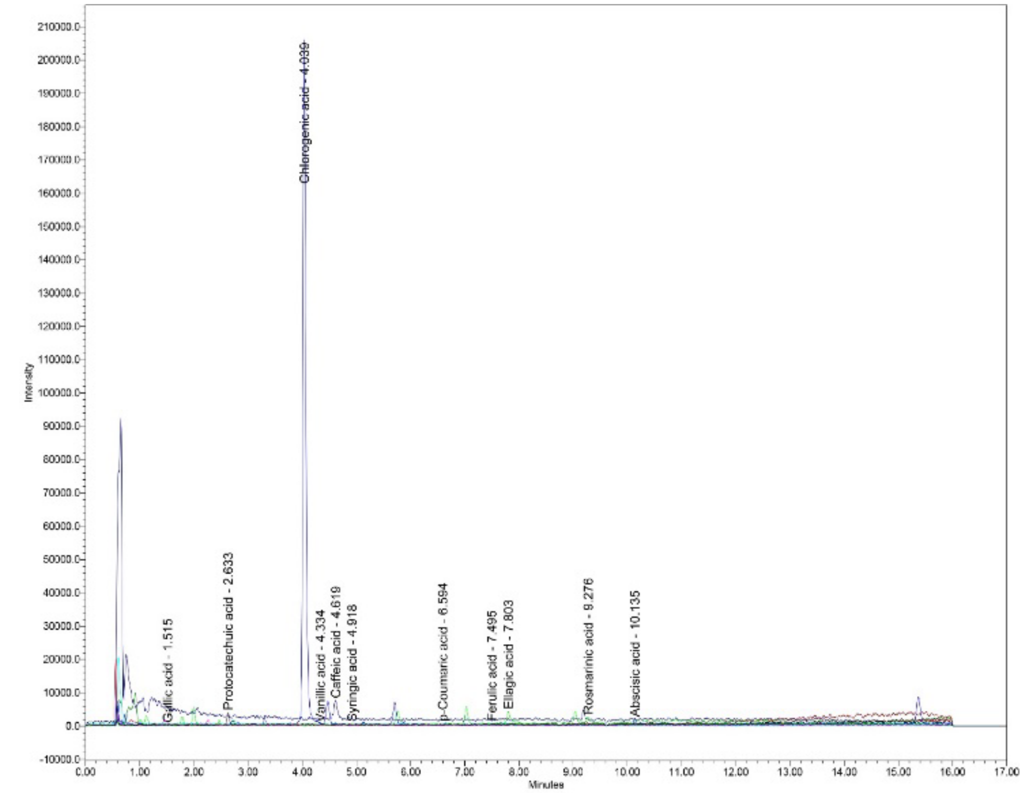

b. *Apii graveolentis* extract – AGE

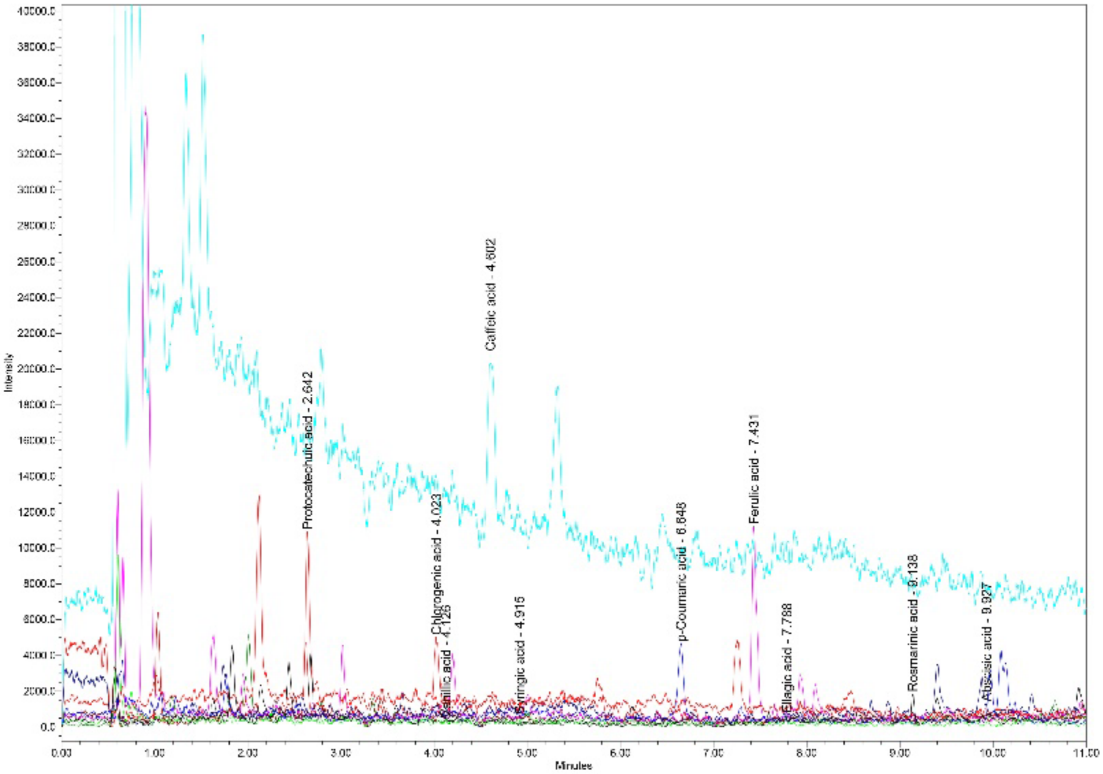

c. *Allii cepae* extract – ACE

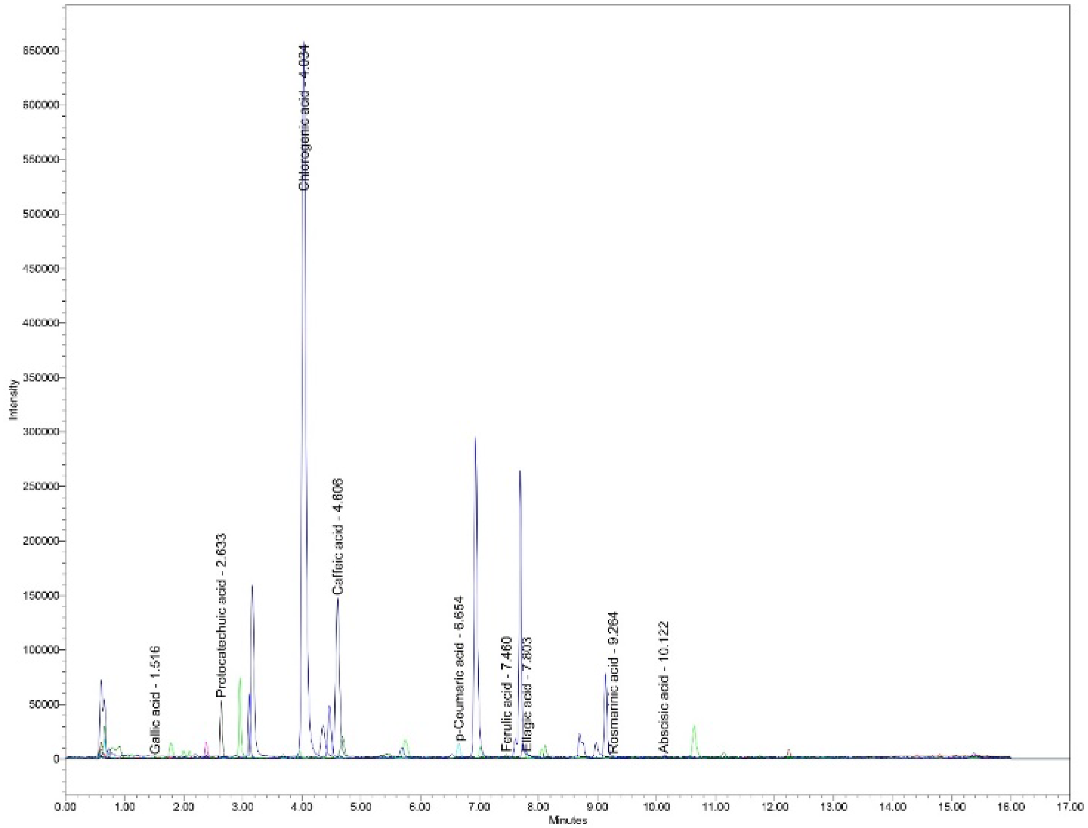

d. *Cichorii* extract – CHE

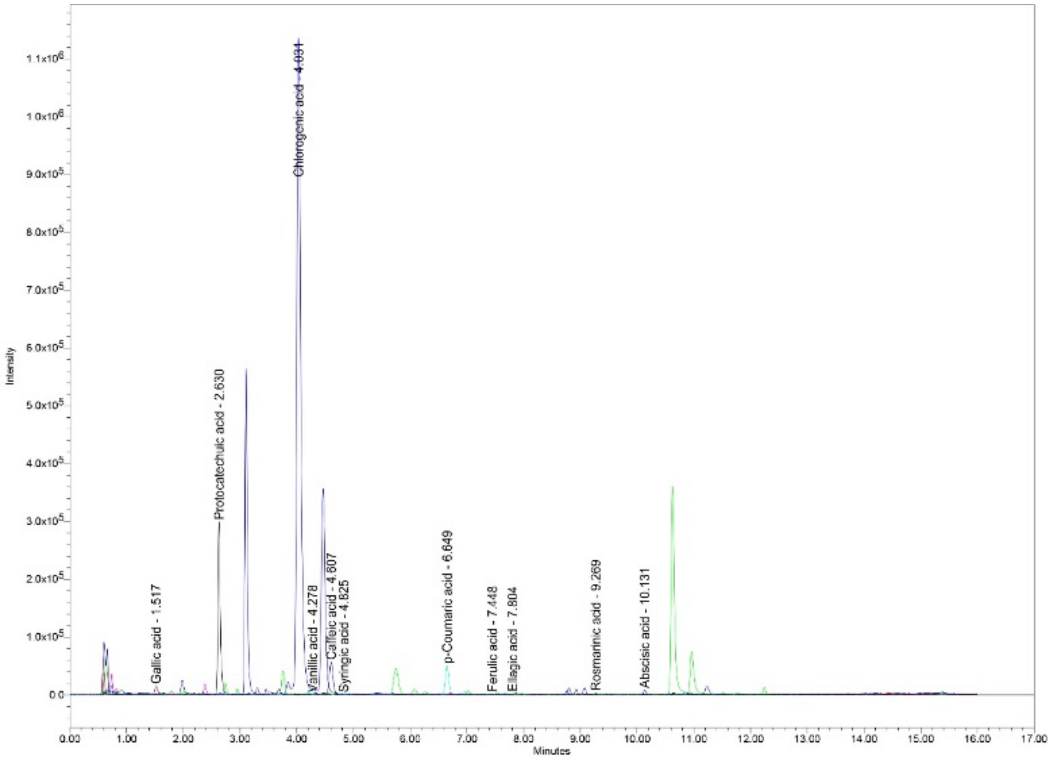

e. *Crataegi* extract – CGE

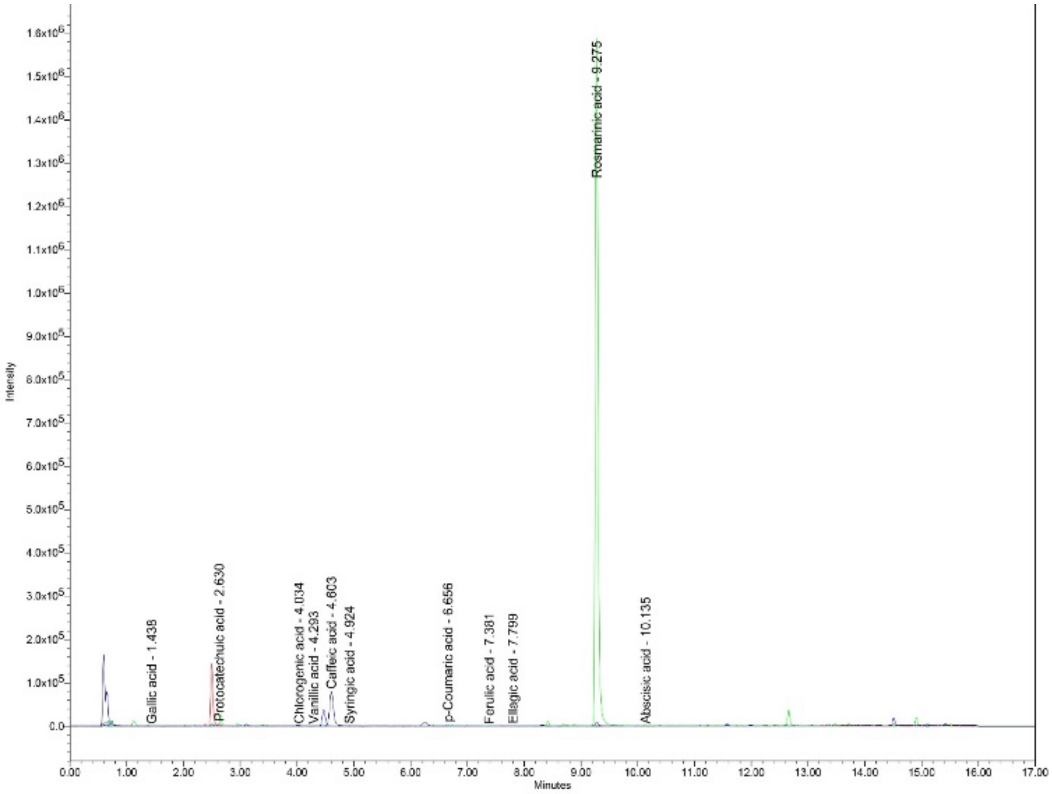

f. *Rosmarini* extract – RSE

Figure S2. The chromatograms obtained using UHPLC–MS method for 6 extracts.

Table S4. Descriptive statistics for DPPH inhibition results

DPPH_Inhibition	N	Mean	Std. Deviation	Std. Error	95% Confidence Interval for Mean		Minimum	Maximum
					Lower Bound	Upper Bound		
DPPH *Allium_cepa*	10	0.1640	0.0175	0.0055	0.1515	0.1765	0.1324	0.1848
DPPH *Rosmarinus*	10	0.5109	0.2038	0.0644	0.3651	0.6567	0.1892	0.7965
DPPH *Cichorium*	10	0.3888	0.1229	0.0389	0.3009	0.4767	0.1972	0.5615
DPPH *Cynara*	10	0.3480	0.1258	0.0398	0.2580	0.4380	0.1742	0.5743
DPPH *Apium*	10	0.1742	0.0242	0.0077	0.1569	0.1916	0.1390	0.2104
DPPH *Crataegus*	10	0.5205	0.2198	0.0695	0.3633	0.6777	0.2096	0.8705
Total	60	0.3511	0.1981	0.0256	0.2999	0.4022	0.1324	0.8705

Table S5. Descriptive statistics for ABTS inhibition results

ABTS_Inhibition	N	Mean	Std. Deviation	Std. Error	95% Confidence Interval for Mean		Minimum	Maximum
					Lower Bound	Upper Bound		
ABTS *Allium_cepa*	7	0.3455	0.0676	0.0255	0.2830	0.4080	0.2343	0.4291
ABTS *Rosmarinus*	7	0.6299	0.1485	0.0561	0.4926	0.7673	0.4174	0.8377
ABTS *Cichorium*	7	0.6073	0.1741	0.0658	0.4463	0.7684	0.3349	0.8177
ABTS *Cynara*	7	0.4348	0.1544	0.0584	0.2920	0.5776	0.1951	0.6255
ABTS *Apium*	7	0.2481	0.0689	0.0260	0.1844	0.3118	0.1514	0.3566
ABTS *Crataegus*	7	0.7249	0.2026	0.0766	0.5375	0.9123	0.4048	0.9505
Total	42	0.4984	0.2183	0.0337	0.4304	0.5664	0.1514	0.9505

Table S6. Descriptive statistics for FRAP optical density results

FRAP_OD	N	Mean	Std. Deviation	Std. Error	95% Confidence Interval for Mean		Minimum	Maximum
					Lower Bound	Upper Bound		
FRAP *Allium_cepa*	10	0.1400	0.0173	0.0055	0.1276	0.1524	0.1183	0.1689
FRAP *Rosmarinus*	10	0.3993	0.1779	0.0563	0.2720	0.5266	0.1444	0.6804
FRAP *Cichorium*	10	0.4602	0.1518	0.0480	0.3517	0.5688	0.2058	0.6695
FRAP *Cynara*	10	0.6385	0.1050	0.0332	0.5634	0.7136	0.4773	0.7909

FRAP *Apium*	10	0.3641	0.0644	0.0204	0.3180	0.4102	0.2590	0.4445
FRAP *Crataegus*	10	0.4859	0.1792	0.0567	0.3577	0.6140	0.2004	0.7346
Total	60	0.4147	0.1964	0.0254	0.3639	0.4654	0.1183	0.7909

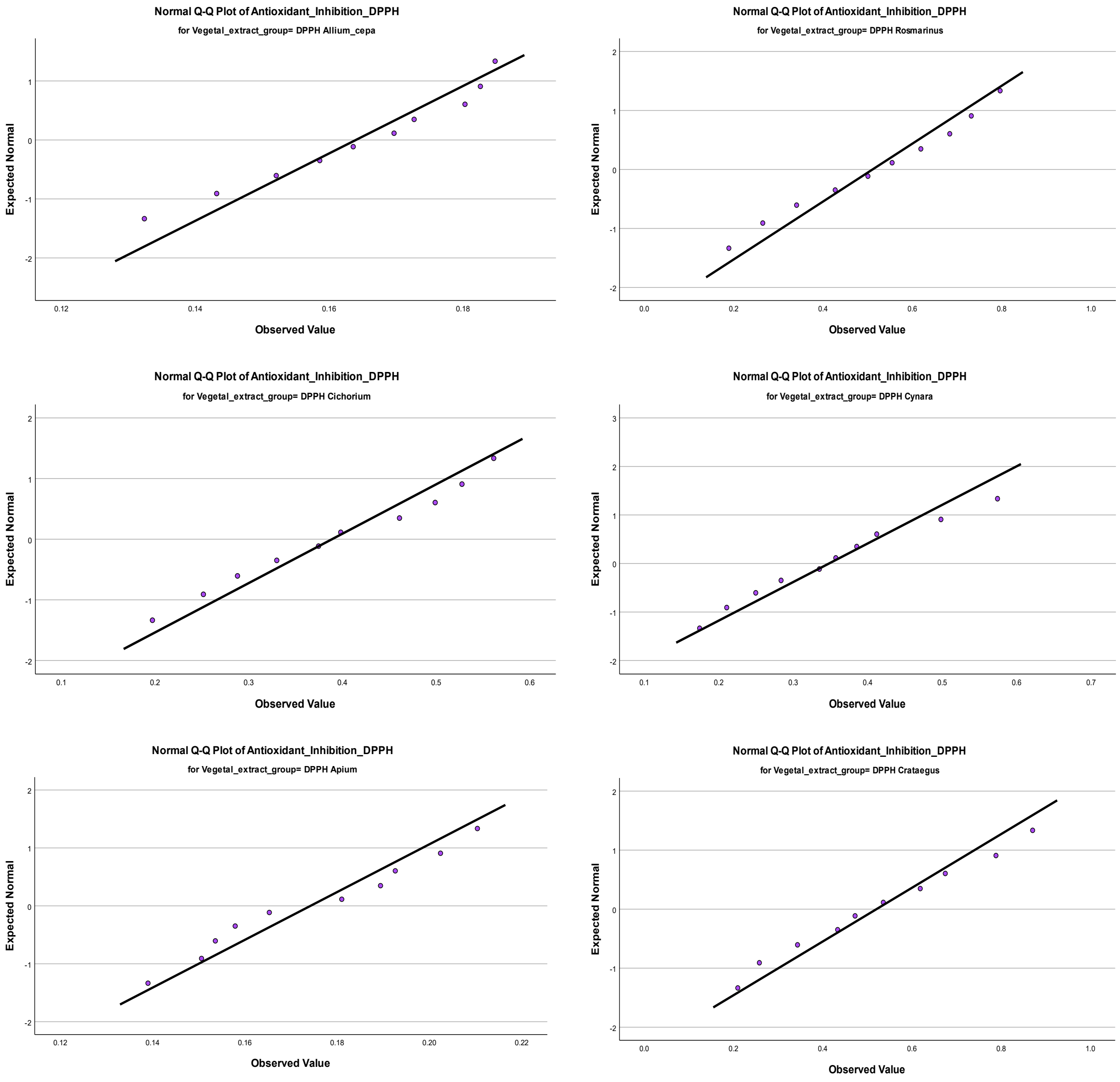

Figure S3. Normal Q-Q Plot for DPPH antioxidant activity

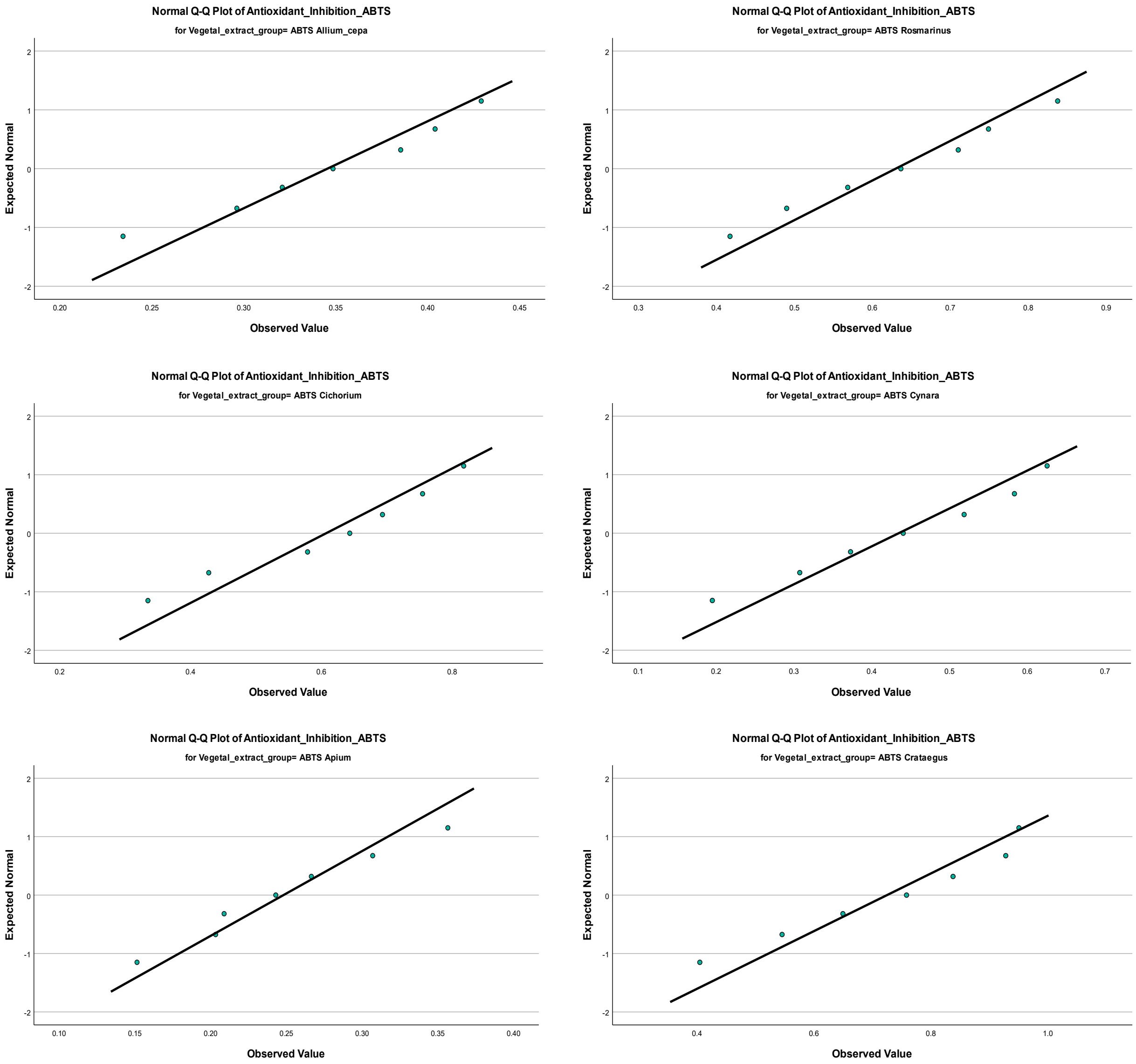

Figure S4. Normal Q-Q Plot for ABTS antioxidant activity

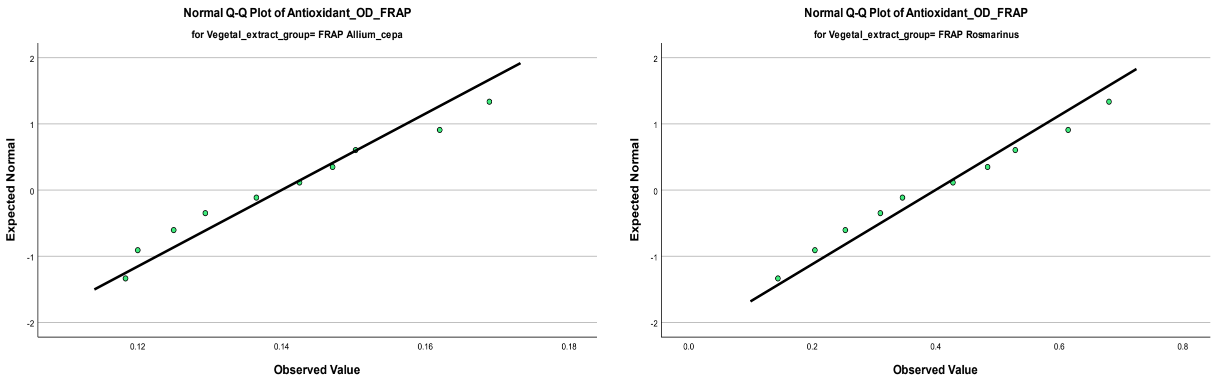

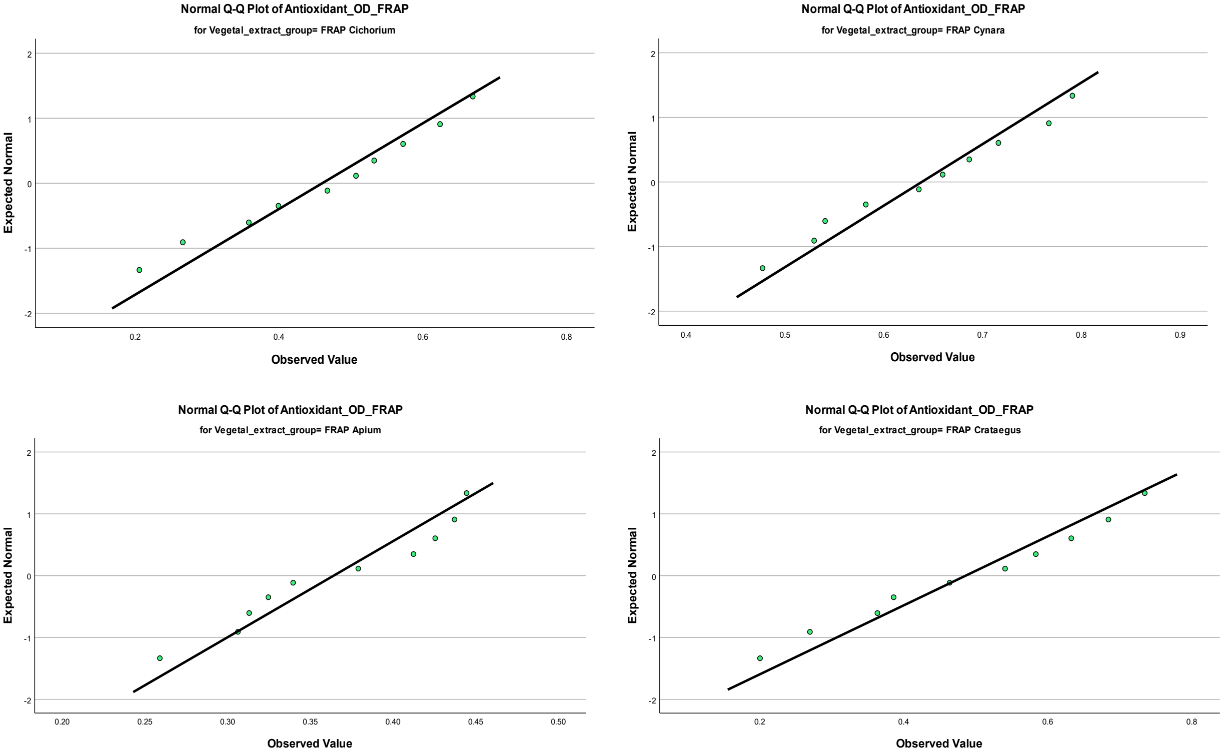

Figure S5. Normal Q-Q Plot for FRAP antioxidant activity

Table S7. ANOVA test for antioxidant DPPH scavenging activity

	Sum of Squares	df	Mean Square	F	Sig.
**Between Groups**	1.219	5	0.244	**12.029**	**<0.001**
**Within Groups**	1.095	54	0.020		
Total	2.314	59			

Table S8. Robust Tests (Antioxidant DPPH)

	Statistic[a]	df1	df2	Sig.
**Welch**	18.843	5	23.264	**<0.001**
**Brown-Forsythe**	12.029	5	29.360	**<0.001**

a. Asymptotically F distributed.

Table S9. Games-Howell Post-Hoc Test for the DPPH method

(I) Vegetal extract group	(J) Vegetal extract group	Mean Difference (I-J)	Std. Error	Sig.	95% Confidence Interval Lower Bound	95% Confidence Interval Upper Bound
DPPH *Allium_cepa*	DPPH *Rosmarinus*	-0.3469*	0.0647	0.004	-0.5759	-0.1178
	DPPH *Cichorium*	-0.2248*	0.0392	0.002	-0.3630	-0.0866
	DPPH *Cynara*	-0.1840*	0.0402	0.011	-0.3255	-0.0425
	DPPH *Apium*	-0.0102	0.0095	0.882	-0.0406	0.0202
	DPPH *Crataegus*	-0.3565*	0.0697	0.006	-0.6034	-0.1096

DPPH *Rosmarinus*	DPPH *Allium_cepa*	0.3469*	0.0647	0.004	0.1178	0.5759
	DPPH *Cichorium*	0.1220	0.0753	0.598	-0.1230	0.3670
	DPPH *Cynara*	0.1629	0.0757	0.315	-0.0832	0.4089
	DPPH *Apium*	0.3366*	0.0649	0.005	0.1075	0.5658
	DPPH *Crataegus*	-0.0096	0.0948	1.000	-0.3110	0.2918
DPPH *Cichorium*	DPPH *Allium_cepa*	0.2248*	0.0392	0.002	0.0866	0.3630
	DPPH *Rosmarinus*	-0.1220	0.0753	0.598	-0.3670	0.1230
	DPPH *Cynara*	0.0408	0.0556	0.975	-0.1359	0.2176
	DPPH *Apium*	0.2146*	0.0396	0.003	0.0762	0.3530
	DPPH *Crataegus*	-0.1317	0.0796	0.580	-0.3925	0.1292
DPPH *Cynara*	DPPH *Allium_cepa*	0.1840*	0.0402	0.011	0.0425	0.3255
	DPPH *Rosmarinus*	-0.1629	0.0757	0.315	-0.4089	0.0832
	DPPH *Cichorium*	-0.0408	0.0556	0.975	-0.2176	0.1359
	DPPH *Apium*	0.1738*	0.0405	0.016	0.0321	0.3155
	DPPH *Crataegus*	-0.1725	0.0801	0.315	-0.4343	0.0893
DPPH *Apium*	DPPH *Allium_cepa*	0.0102	0.0095	0.882	-0.0202	0.0406
	DPPH *Rosmarinus*	-0.3366*	0.0649	0.005	-0.5658	-0.1075
	DPPH *Cichorium*	-0.2146*	0.0396	0.003	-0.3530	-0.0762
	DPPH *Cynara*	-0.1738*	0.0405	0.016	-0.3155	-0.0321
	DPPH *Crataegus*	-0.3463*	0.0699	0.007	-0.5933	-0.0992
DPPH C*rataegus*	DPPH *Allium_cepa*	0.3565*	0.0697	0.006	0.1096	0.6034
	DPPH *Rosmarinus*	0.0096	0.0948	1.000	-0.2918	0.3110
	DPPH *Cichorium*	0.1317	0.0796	0.580	-0.1292	0.3925
	DPPH *Cynara*	0.1725	0.0801	0.315	-0.0893	0.4343
	DPPH *Apium*	0.3463*	0.0699	0.007	0.0992	0.5933

*. The mean difference is significant at the 0.05 level.

Table S10. ANOVA test for antioxidant ABTS scavenging activity

	Sum of Squares	df	Mean Square	F	Sig.
**Between Groups**	1.194	5	0.239	**11.316**	**<0.001**
**Within Groups**	0.760	36	0.021		
Total	1.953	41			

Table S11. Robust Tests (Antioxidant ABTS)

	Statistic[a]	df1	df2	Sig.
**Welch**	13.744	5	16.292	**<0.001**
**Brown-Forsythe**	11.316	5	25.965	**<0.001**

a. Asymptotically F distributed.

Table S12. Games-Howell Post-Hoc Test for the ABTS method

(I) Vegetal extract group	(J) Vegetal extract group	Mean Difference (I-J)	Std. Error	Sig.	95% Confidence Interval	
					Lower Bound	Upper Bound
ABTS *Allium_cepa*	ABTS *Rosmarinus*	-0.2844*	0.0617	0.013	-0.5072	-0.0617
	ABTS *Cichorium*	-0.2619*	0.0706	0.048	-0.5218	-0.0019

	ABTS *Cynara*	-0.0893	0.0637	0.726	-0.3206	0.1419
	ABTS *Apium*	0.0974	0.0365	0.153	-0.0252	0.2199
	ABTS *Crataegus*	-0.3794*	0.0807	0.016	-0.6815	-0.0774
ABTS *Rosmarinus*	ABTS *Allium_cepa*	0.2844*	0.0617	0.013	0.0617	0.5072
	ABTS *Cichorium*	0.0226	0.0865	1.000	-0.2692	0.3144
	ABTS *Cynara*	0.1951	0.0810	0.227	-0.0770	0.4672
	ABTS *Apium*	0.3818*	0.0619	0.002	0.1589	0.6048
	ABTS *Crataegus*	-0.0950	0.0950	0.908	-0.4188	0.2288
ABTS *Cichorium*	ABTS *Allium_cepa*	0.2619*	0.0706	0.048	0.0019	0.5218
	ABTS *Rosmarinus*	-0.0226	0.0865	1.000	-0.3144	0.2692
	ABTS *Cynara*	0.1725	0.0880	0.415	-0.1237	0.4687
	ABTS *Apium*	0.3592*	0.0708	0.009	0.0992	0.6193
	ABTS *Crataegus*	-0.1176	0.1010	0.845	-0.4581	0.2229
ABTS *Cynara*	ABTS *Allium_cepa*	0.0893	0.0637	0.726	-0.1419	0.3206
	ABTS *Rosmarinus*	-0.1951	0.0810	0.227	-0.4672	0.0770
	ABTS *Cichorium*	-0.1725	0.0880	0.415	-0.4687	0.1237
	ABTS *Apium*	0.1867	0.0639	0.129	-0.0447	0.4181
	ABTS *Crataegus*	-0.2901	0.0963	0.093	-0.6174	0.0372
ABTS *Apium*	ABTS *Allium_cepa*	-0.0974	0.0365	0.153	-0.2199	0.0252
	ABTS *Rosmarinus*	-0.3818*	0.0619	0.002	-0.6048	-0.1589
	ABTS *Cichorium*	-0.3592*	0.0708	0.009	-0.6193	-0.0992
	ABTS *Cynara*	-0.1867	0.0639	0.129	-0.4181	0.0447
	ABTS *Crataegus*	-0.4768*	0.0809	0.004	-0.7789	-0.1747
ABTS C*rataegus*	ABTS *Allium_cepa*	0.3794*	0.0807	0.016	0.0774	0.6815
	ABTS *Rosmarinus*	0.0950	0.0950	0.908	-0.2288	0.4188
	ABTS *Cichorium*	0.1176	0.1010	0.845	-0.2229	0.4581
	ABTS *Cynara*	0.2901	0.0963	0.093	-0.0372	0.6174
	ABTS *Apium*	0.4768*	0.0809	0.004	0.1747	0.7789

*. The mean difference is significant at the 0.05 level.

Table S13. ANOVA test for antioxidant FRAP scavenging activity

	Sum of Squares	df	Mean Square	F	Sig.
**Between Groups**	1.355	5	0.271	**15.897**	**<0.001**
**Within Groups**	0.920	54	0.017		
Total	2.275	59			

Table S14. Robust Tests (Antioxidant FRAP)

	Statistic[a]	df1	df2	Sig.
**Welch**	71.918	5	21.939	**<0.001**
**Brown-Forsythe**	15.897	5	34.829	**<0.001**

a. Asymptotically F distributed.

Table S15. Games-Howell Post-Hoc Test for the FRAP method

(I) Vegetal extract group	(J) Vegetal extract group	Mean Difference (I-J)	Std. Error	Sig.	95% Confidence Interval Lower Bound	95% Confidence Interval Upper Bound
FRAP *Allium_cepa*	FRAP *Rosmarinus*	-0.2593*	0.0565	0.011	-0.4593	-0.0594
	FRAP *Cichorium*	-0.3202*	0.0483	0.000	-0.4908	-0.1496
	FRAP *Cynara*	-0.4985*	0.0336	0.000	-0.6166	-0.3803
	FRAP *Apium*	-0.2241*	0.0211	0.000	-0.2969	-0.1513
	FRAP *Crataegus*	-0.3459*	0.0569	0.002	-0.5472	-0.1445
FRAP *Rosmarinus*	FRAP *Allium_cepa*	0.2593*	0.0565	0.011	0.0594	0.4593
	FRAP *Cichorium*	-0.0609	0.0740	0.959	-0.2965	0.1748
	FRAP *Cynara*	-0.2391*	0.0653	0.024	-0.4522	-0.0261
	FRAP *Apium*	0.0352	0.0598	0.990	-0.1678	0.2383
	FRAP *Crataegus*	-0.0865	0.0799	0.882	-0.3403	0.1673
FRAP *Cichorium*	FRAP *Allium_cepa*	0.3202*	0.0483	0.000	0.1496	0.4908
	FRAP *Rosmarinus*	0.0609	0.0740	0.959	-0.1748	0.2965
	FRAP *Cynara*	-0.1783	0.0584	0.068	-0.3663	0.0097
	FRAP *Apium*	0.0961	0.0521	0.476	-0.0787	0.2709
	FRAP *Crataegus*	-0.0257	0.0743	0.999	-0.2623	0.2110
FRAP *Cynara*	FRAP *Allium_cepa*	0.4985*	0.0336	0.000	0.3803	0.6166
	FRAP *Rosmarinus*	0.2391*	0.0653	0.024	0.0261	0.4522
	FRAP *Cichorium*	0.1783	0.0584	0.068	-0.0097	0.3663
	FRAP *Apium*	0.2744*	0.0389	0.000	0.1478	0.4010
	FRAP *Crataegus*	0.1526	0.0657	0.246	-0.0617	0.3669
FRAP *Apium*	FRAP *Allium_cepa*	0.2241*	0.0211	0.000	0.1513	0.2969
	FRAP *Rosmarinus*	-0.0352	0.0598	0.990	-0.2383	0.1678
	FRAP *Cichorium*	-0.0961	0.0521	0.476	-0.2709	0.0787
	FRAP *Cynara*	-0.2744*	0.0389	0.000	-0.4010	-0.1478
	FRAP *Crataegus*	-0.1218	0.0602	0.387	-0.3262	0.0826
FRAP C*rataegus*	FRAP *Allium_cepa*	0.3459*	0.0569	0.002	0.1445	0.5472
	FRAP *Rosmarinus*	0.0865	0.0799	0.882	-0.1673	0.3403
	FRAP *Cichorium*	0.0257	0.0743	0.999	-0.2110	0.2623
	FRAP *Cynara*	-0.1526	0.0657	0.246	-0.3669	0.0617
	FRAP *Apium*	0.1218	0.0602	0.387	-0.0826	0.3262

*. The mean difference is significant at the 0.05 level.

Table S16. The chemical content and antioxidant values of plant extracts

Plant extract	FL (g rutoside/100 g DW)	TP (g tannic acid/100 g DW)	PCA (g chlorogenic acid/100 g DW)	IC50 ABTS (mg/mL)	IC50 DPPH (mg/mL)	EC50 FRAP (mg/mL)
ACE	2.03	3.77	ND	0.18	1.32	1.41
RSE	3.71	39.62	22.05	0.04	0.11	0.15
CHE	3.64	30.51	6.16	0.15	0.34	0.26
CE	4.38	7.74	4.47	0.20	0.37	0.06

AGE	0.35	0.57	0.38	2.66	10.31	2.65
CGE	5.32	25.93	14.05	0.03	0.11	0.13

ND – not detected.

Table S17. Correlations among variables

	**INVSQRT_ ABTS_IC50**	**INVSQRT_ DPPH_IC50**	**INVSQRT_FRAP _EC50**	**SQRT_ TP**	**SQRT_ PCA**	**FL_value**
**INVSQRT_ ABTS_IC50**	1					
**INVSQRT_ DPPH_IC50**	0.961**	1				
**INVSQRT_ FRAP_EC50**	0.474	0.640	1			
**SQRT_TP**	−0.825*	−0.895*	−0.474	1		
**SQRT_PCA**	−0.850*	−0.951**	−0.626	0.893*	1	
**FL_value**	−0.774	−0.841*	−0.841*	0.733	0.732	1

**. $p < 0.01$ - Correlation is significant at the 0.01 level (2-tailed).

*. $p < 0.05$ - Correlation is significant at the 0.05 level (2-tailed). $N = 6$

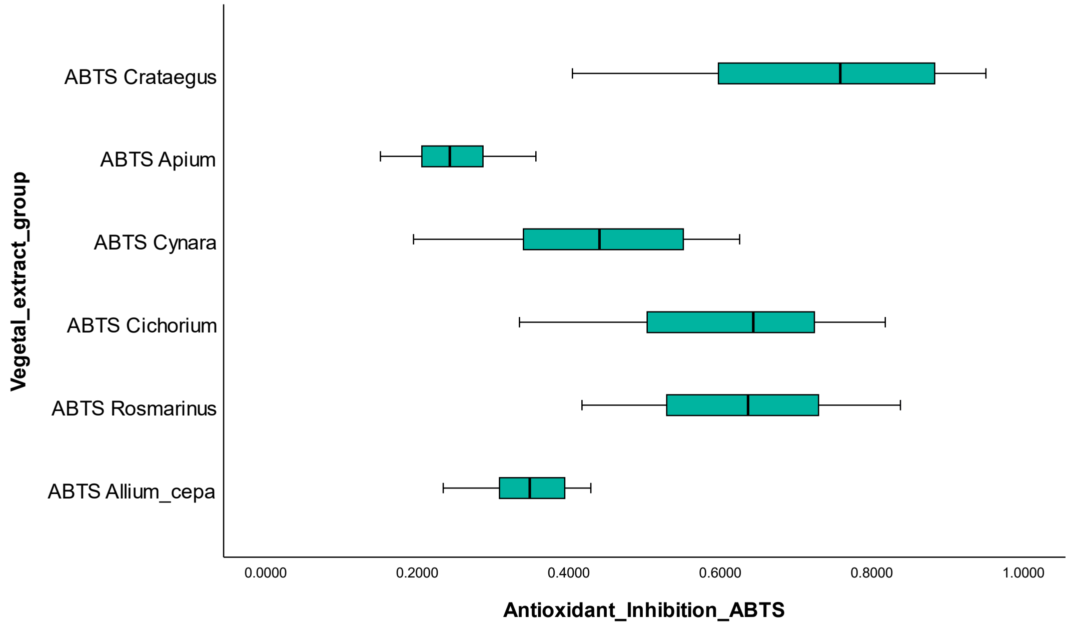

Figure S6. The boxplot distribution of ABTS antioxidant inhibition within groups

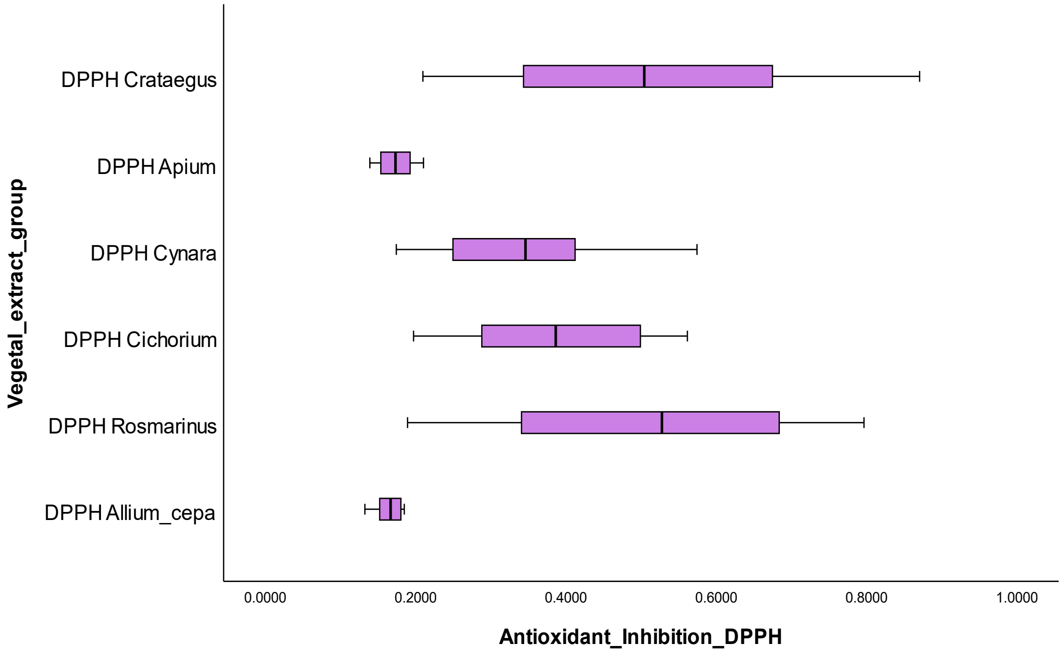

Figure S7. The boxplot distribution of DPPH antioxidant inhibition within groups

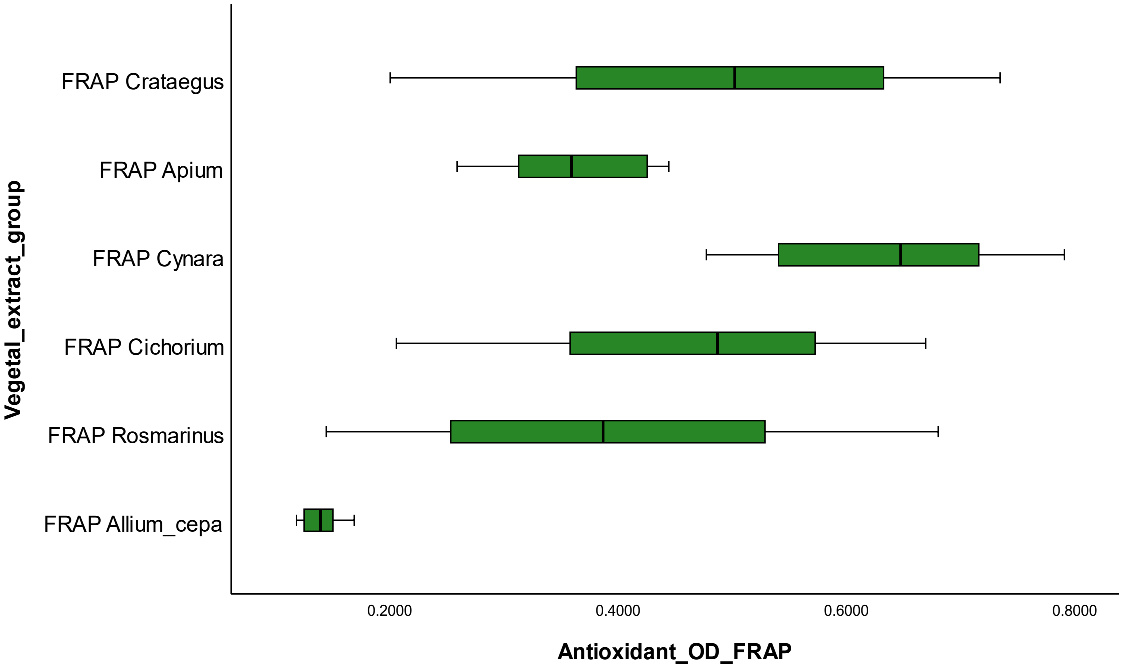

Figure S8. The boxplot distribution of FRAP optical density within groups

Table S18. Activity prediction results using PASS algorithm

Activity	Probability	
	**Rosmarinic acid**	**Chlorogenic acid**
Antioxidant	0.539	0.785
Reductant	-	0.555
Free radical scavenger	0.745	0.856
Lipid peroxidase inhibitor	0.719	0.855
Hypolipidemic	0.699	0.570
Lipid metabolism regulator	-	0.565
Cholesterol antagonist	0.570	-
Peroxisome proliferator-activated receptor agonist	0.547	-

Table S19. Target prediction using SwissTargetPrediction and SEA

Target	Rosmarinic acid		Chlorogenic acid		Acetazolamide	
	**Probability***	**MaxTC****	**Probability***	**MaxTC****	**Probability***	**MaxTC****
Carbonic anhydrase I	0.1093	-	0.0972	-	0.3180	1.0000
Carbonic anhydrase II	-	-	0.0972	-	0.3180	1.0000
Carbonic anhydrase III	-	0.5000	-	-	0.2915	1.0000
Carbonic anhydrase IV	0.1093	0.7600	-	-	0.1948	1.0000
Carbonic anhydrase VA	-	0.5000		0.4468	0.2915	1.0000
Carbonic anhydrase VB	0.1093	0.5000	0.0972	0.4468	0.2915	1.0000
Carbonic anhydrase VI	0.1093	0.5000	-	0.4468	0.2915	1.0000
Carbonic anhydrase VII	0.1093	0.7600	-	0.4468	0.1333	1.0000
Carbonic anhydrase IX	0.1093	-	0.0972	-	0.3180	1.0000
Carbonic anhydrase XII	0.1256	-	0.0972	-	0.3180	1.0000
Carbonic anhydrase XIV	0.1093	0.5000	-	0.4468	0.1948	1.0000

* - calculated with SwissTargetPrediction;

** - calculated with SEA; MaxTC - maximum Tanimoto coefficient.

Table S20. Quality parameters for the human CA5A homology models

Parameter	Template	
	**1KEQ (1.88 Å)**	**1DMY (2.45 Å)**
Clashscore, all atoms:	1	1.24
Poor rotamers	3 (1.37%)	1 (0.46%)
Favored rotamers	211 (96.35%)	214 (97.72%)
Ramachandran outliers	0	0
Ramachandran favored	247 (97.63%)	248 (98.02%)
Z score	-0.74 ± 0.43	0.14 ± 0.47
MolProbity score	0.98	0.84
Cβ deviations > 0.25 Å	0	0
Bad bonds	8 / 2109 (0.38%)	4 / 2106 (0.19%)
Bad angles	17 / 2875 (0.59%)	17 / 2878 (0.59%)

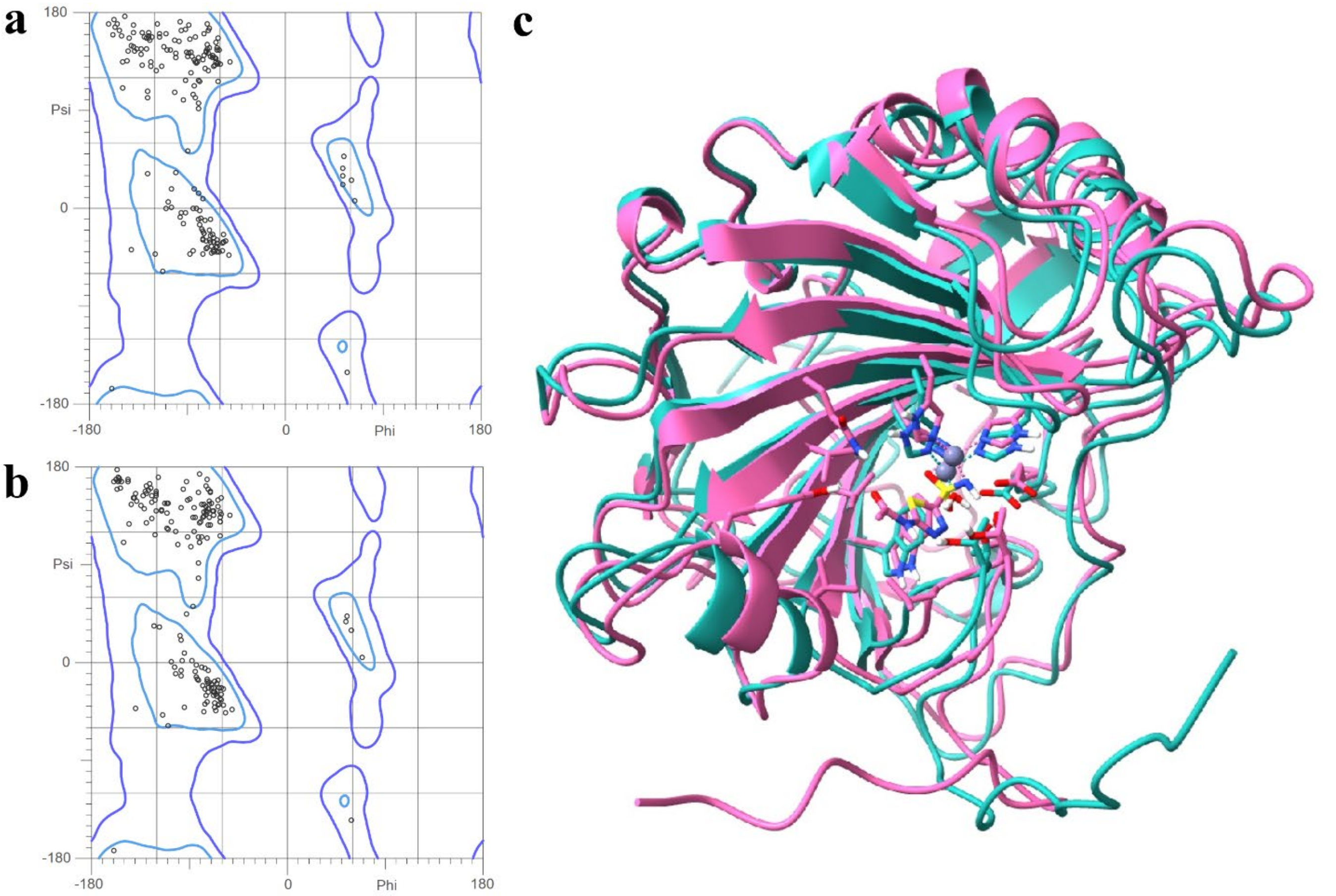

Figure S9. Homology modeling results for human CA5A. (**a**) Ramachandran plot after using the 1KEQ template; (**b**) Ramachandran plot after using the 1DMY template; (**c**) Superposition between the two homology models (cyan – 1KEQ template, magenta – 1DMY template).

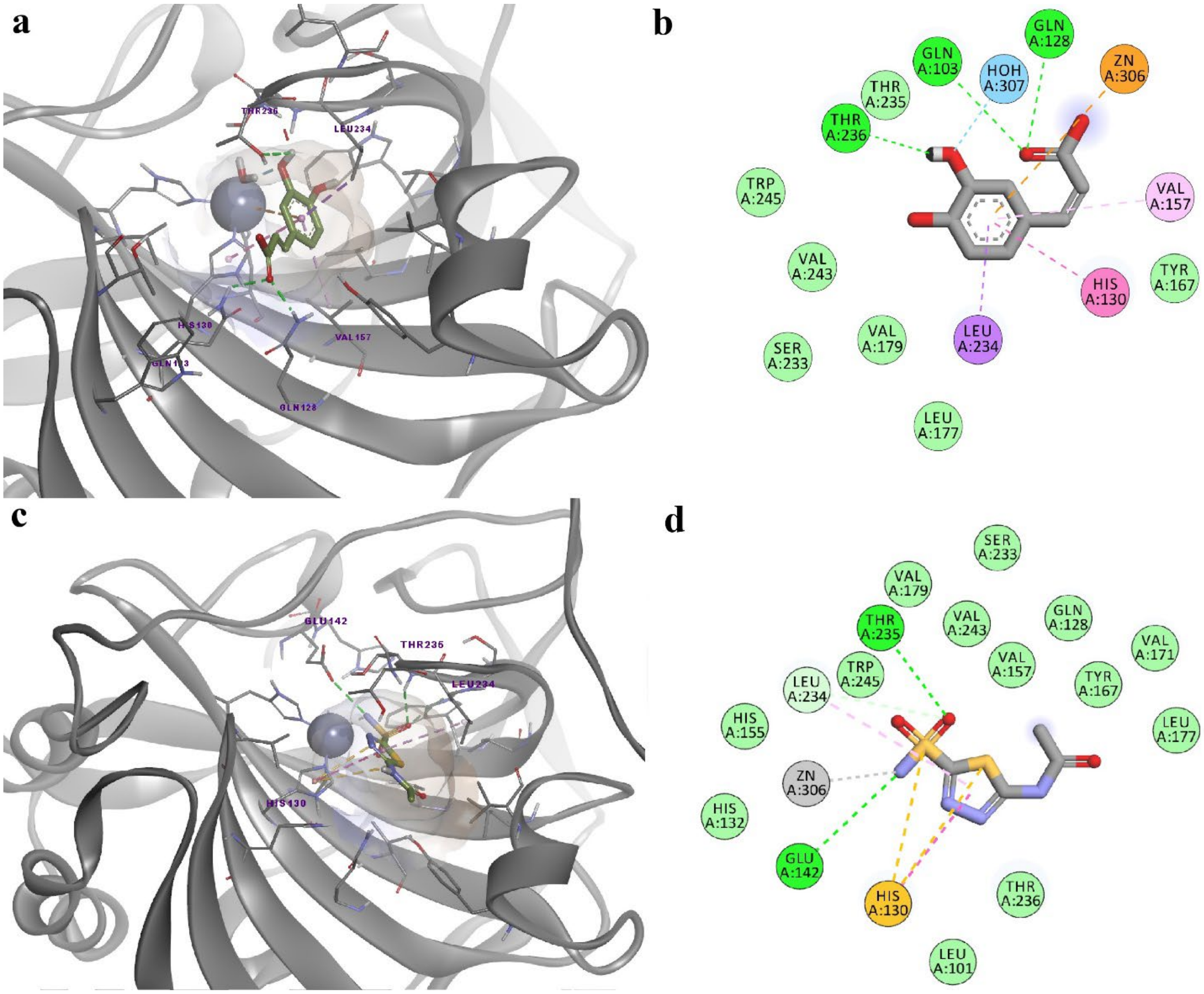

Figure S10. Predicted binding poses of positive controls CFA and AZM in CA5A active site. (**a**) predicted conformation of CFA-CA5A complex; (**b**) 2D diagram of predicted interactions between CFA and CA5A; (**c**) predicted conformation of AZM-CA5A complex; (**d**) 2D diagram of predicted interactions between AZM and CA5A. Green dashes – hydrogen bonds, blue dashes – hydrogen bond with water molecules, grey dashes – metal interaction. orange dashes – attractive charges, purple dashes – pi-sigma interactions, magenta dashes – pi-pi T-shaped interactions, pink dashes – pi-alkyl interactions, yellow dashes – pi-sulfur interaction, light green circles – van der Waals interactions.

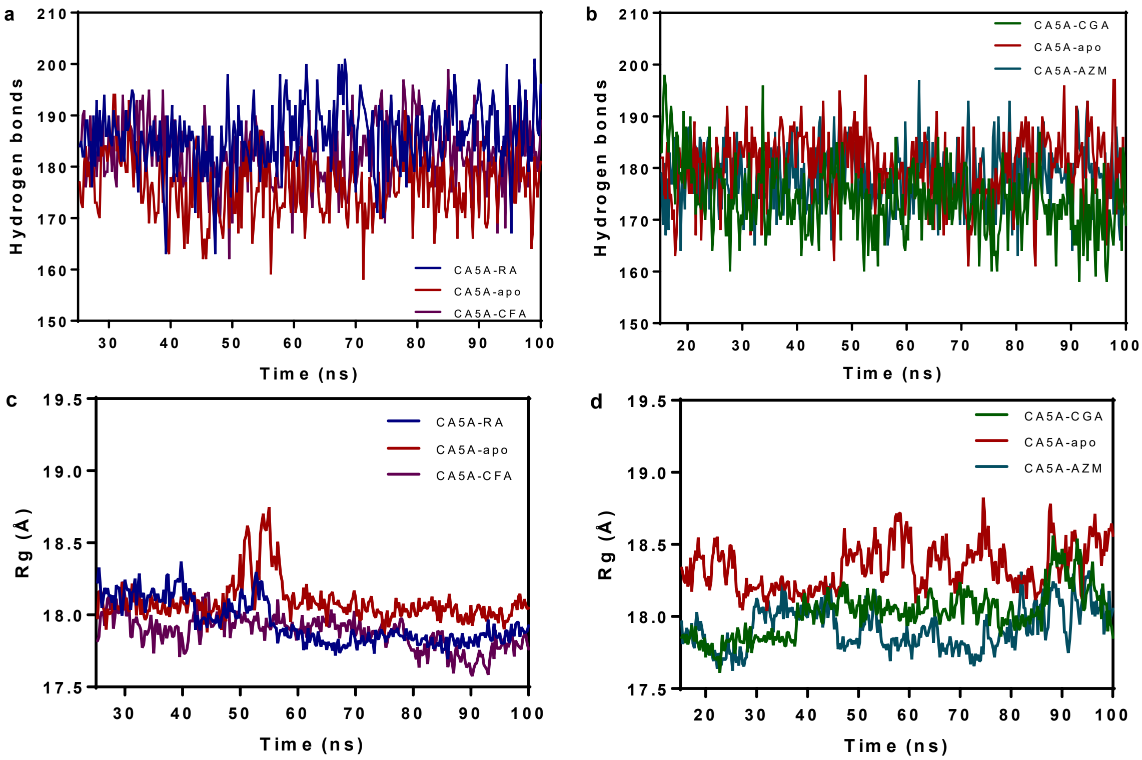

Figure S11. Results following 100 ns MD simulation. (**a**) Number of intramolecular hydrogen bonds for CA5A-RA complex vs controls; (**b**) Number of intramolecular hydrogen bonds for CA5A-CGA complex vs controls; (**c**) Radius of gyration (Rg) for CA5A-RA complex vs controls; (**d**) Radius of gyration for CA5A-CGA vs controls.
